# Reprogramming bacterial protein organelles as a nanoreactor for hydrogen production

**DOI:** 10.1038/s41467-020-19280-0

**Published:** 2020-10-28

**Authors:** Tianpei Li, Qiuyao Jiang, Jiafeng Huang, Catherine M. Aitchison, Fang Huang, Mengru Yang, Gregory F. Dykes, Hai-Lun He, Qiang Wang, Reiner Sebastian Sprick, Andrew I. Cooper, Lu-Ning Liu

**Affiliations:** 1 Institute of Systems, Molecular and Integrative Biology, University of Liverpool, Liverpool, L69 7ZB UK; 2 Key Laboratory of Algal Biology, Institute of Hydrobiology, Chinese Academy of Sciences, 430072 Wuhan, China; 3 University of Chinese Academy of Sciences, 100049 Beijing, China; 4 State Key Laboratory of Crop Stress Adaptation and Improvement, School of Life Sciences, Henan University, 475004 Kaifeng, China; 5 School of Life Sciences, Central South University, Changsha, China; 6 Materials Innovation Factory and Department of Chemistry, University of Liverpool, Liverpool, L7 3NY UK; 7 Innovation Academy for Seed Design, Chinese Academy of Sciences, Beijing, China; 8 College of Marine Life Sciences, and Frontiers Science Center for Deep Ocean Multispheres and Earth System, Ocean University of China, 266003 Qingdao, China

**Keywords:** Biocatalysis, Synthetic biology, Biosynthesis, Bioenergy

## Abstract

Compartmentalization is a ubiquitous building principle in cells, which permits segregation of biological elements and reactions. The carboxysome is a specialized bacterial organelle that encapsulates enzymes into a virus-like protein shell and plays essential roles in photosynthetic carbon fixation. The naturally designed architecture, semi-permeability, and catalytic improvement of carboxysomes have inspired rational design and engineering of new nanomaterials to incorporate desired enzymes into the protein shell for enhanced catalytic performance. Here, we build large, intact carboxysome shells (over 90 nm in diameter) in the industrial microorganism *Escherichia coli* by expressing a set of carboxysome protein-encoding genes. We develop strategies for enzyme activation, shell self-assembly, and cargo encapsulation to construct a robust nanoreactor that incorporates catalytically active [FeFe]-hydrogenases and functional partners within the empty shell for the production of hydrogen. We show that shell encapsulation and the internal microenvironment of the new catalyst facilitate hydrogen production of the encapsulated oxygen-sensitive hydrogenases. The study provides insights into the assembly and formation of carboxysomes and paves the way for engineering carboxysome shell-based nanoreactors to recruit specific enzymes for diverse catalytic reactions.

## Abstract (web summary)

The extreme oxygen sensitive character of hydrogenases is a longstanding issue for hydrogen production in bacteria. Here, the authors build carboxysome shells in *E. coli* and incorporate catalytically active hydrogenases and functional partners within the empty shell for the production of hydrogen.

## Introduction

Due to climate change, there is a pressing need to reduce the emission of carbon dioxide from burning fossil fuels. Hydrogen has been identified as a potential replacement for sustainable and clean energy sources, since consuming hydrogen generates only water as the byproduct and the high-energy content of the H-H bond making hydrogen an efficient means of storing energy^1,2^. However, most hydrogen is currently produced non-renewably from steam-reforming processes, which release a substantial amount of carbon dioxide^3^. Therefore, increasing attention has been paid to the development of novel hydrogen-forming catalysts in chemistry and biology^4^.

Hydrogenases are enzymes that catalyze the generation and conversion of hydrogen. The exceptional catalytic activity and efficiency of hydrogenases make them valid candidates for biological hydrogen production in synthetic engineering^5^. Three distinct classes of hydrogenases have been discovered: [Fe]-hydrogenases, [NiFe]-hydrogenases and [FeFe]-hydrogenases^6^. Among these hydrogenases, [FeFe]-hydrogenases are the most efficient enzymes for catalytic hydrogen turnover, and arguably the promising biocatalysts for hydrogen production. However, a major drawback of [FeFe]-hydrogenases is their extreme oxygen sensitivity and irreversible inactivation by oxygen^7,8^. To overcome these limitations, an innovative strategy is to incorporate hydrogenases to synthetic scaffolding platforms, such as polymersomes, liposomes and virus-like particles, ensuring the encapsulation and condensation of enzymes as well as the modulation of enzymatic activities^9,10^. It has been recently shown that the protein-based capsid of bacteriophage P22 could encapsulate oxygen-tolerant [NiFe]-hydrogenases and offer stability and protection to the enzymes; the elevated local concentration of hydrogenases in the nanoreactor led to improved hydrogen-producing activities^11^. This represents a step towards synthetically engineering large and stable nanoreactors that are capable of sequestering high copy numbers of cargo enzymes and providing selective permeability to substrates to boost enzyme activities.

**Fig. 1:**
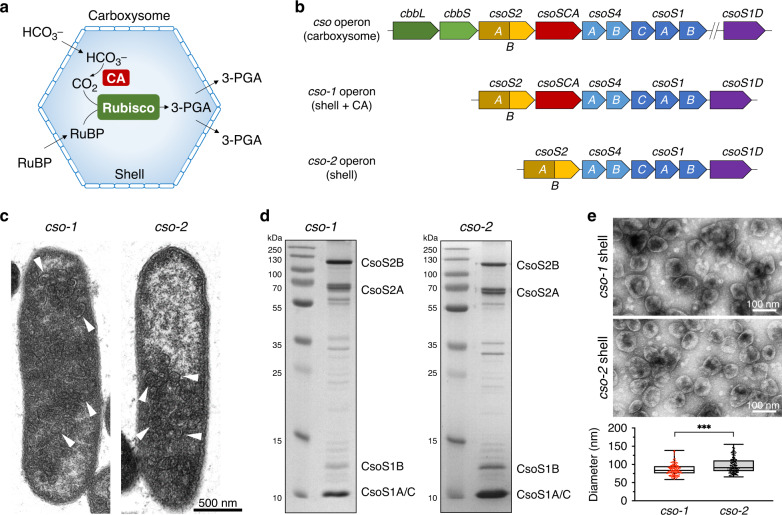
Engineering and characterization of empty α-carboxysome shells. **a** Schematic of the carboxysome structure and metabolic pathways. The carboxysome shell serves as a physical barrier to permit passage of cytosolic bicarbonate (HCO_3_^−^) and ribulose-1,5-bisphosphate (RuBP) into the carboxysome. The metabolic product 3-phosphoglycerate (3-PGA) is transported across the shell and is metabolized via the Calvin–Benson–Bassham cycle. **b** Genetic organizations of the native *cso* operon, synthetic *cso-1* and *cso-2* operons. **c** Thin-section electron microscopy (EM) of *E. coli* cells expressing the *cso-1* operon (left) and *cso-2* operon (right), respectively. Arrows indicate α-carboxysome shell particles discerned in the cell. **d** SDS-PAGE of purified *cso-1* (left) and *cso-2* (right) shell structures. **e** Transmission EM of purified *cso-1* (top) and *cso-2* (middle) shells in the 20% sucrose fractions. Shells with CA are significantly smaller in diameter than those without CA. ***p < 0.001 (*p* = 0.0007, *n* = 150, two-tailed unpaired *t*-test), implying the role of CA in confining shell architecture (bottom). Box and whisker plots indicate the median (middle line in the box), 25th percentile (bottom line of the box), 75th percentile (top line of the box), as well as the minima and maxima (whiskers). Source data underlying **c–e** are provided as a Source data file.

In nature, many bacteria have evolved virus-like proteinaceous organelles, termed bacterial microcompartments (BMCs), to compartmentalize metabolic reactions and confines toxic/inhibitory metabolites to mitigate the crosstalk of metabolites^12–14^. BMCs encapsulate catalytic enzymes within an icosahedral shell that serves as a physical barrier to selectively mediate flux of metabolites, while condensing and protecting cargo enzymes^15–17^. Carboxysomes (including α-carboxysomes and β-carboxysomes) are anabolic BMCs, functioning as the key CO_2_-fixing machinery in all cyanobacteria and some chemoautotrophs^18–21^. The primary carboxylating enzymes, ribulose-1,5-bisphosphate carboxylase oxygenase (Rubisco), and carbonic anhydrase (CA), are encased by the carboxysome shell that is constructed by diverse shell proteins through self-assembly^21–24^. The shell allows entry of HCO_3_^−^ into the carboxysome interior, where CA dehydrates HCO_3_^−^ into CO_2_ in the vicinity of Rubisco enzymes (Fig. 1a). The carboxysome shell is also believed to exclude passage of O_2_ and reduce CO_2_ leakage into the cytosol^25^, resulting in a CO_2_-rich microenvironment that favours the carboxylation of Rubisco, thereby enhancing carbon fixation. The intriguing features of carboxysomes—self-assembly, modularity, shell permeability, and catalytic enhancement—have made them attractive engineering objectives to supercharge cellular metabolism^26^. Some of these principles have been incorporated into synthetic, abiotic materials such as metal-organic frameworks to allow cooperative absorption of CO_2_^27^.

We reasoned that the naturally occurring microenvironment generated within the carboxysome shell might enhance the catalytic performance of [FeFe]-hydrogenases, and in general any O_2_-sensitive enzymes and molecules. Thus, rewiring the carboxysomal shell to encapsulate [FeFe]-hydrogenases shows promise for creating a catalyst to integrate hydrogen-producing enzymes and strengthen their oxygen tolerance for improving hydrogen production. This notion has been supported by the facts that recombinant carboxysome structures could be engineered and modulated in non-native hosts, such as *Escherichia coli*^28,29^.

Here, we generate the heterologous expression systems to produce intact, stable α-carboxysome shells (~100 nm in diameter) in *E. coli* and then perform systematic characterizations of the assembly of the protein shells. We also define the endogenous encapsulation peptide (EP) to direct external proteins into the shell. Based on the developed expression and encapsulation systems, we recruit [FeFe]-hydrogenases and their partners ferredoxin (Fd) from the green alga *Chlamydomonas reinhardtii* and ferredoxin:NADP^+^-oxidoreductase (FNR) from *E. coli* into the shell, to create a catalytically functional hydrogen-producing nanoreactor in *E. coli*. Compared to free hydrogenases, this hydrogenase-encapsulating catalyst possesses an improved performance for H_2_ production and also has superior to O_2_ resistance. Our study provides insight into the self-assembly and selective permeability of α-carboxysomes, which is extendable to other protein organelles. It also informs bioinspired design and engineering of metabolic nanoreactors for a diverse range of functions.

## Results and discussion

### Generation of an entire synthetic α-carboxysome shell

According to the types of encapsulated Rubisco, carboxysomes can be categorized into α-carboxysomes and β-carboxysomes^19,30^. Distinct from the ‘inside out’ de novo assembly of β-carboxysomes^31–33^, the assembly of α-carboxysomes appears to start from shell formation^34^ or a simultaneous shell-interior assembly^35^, highlighting the possibility of generating and reprogramming entire α-carboxysome shells.

The α-carboxysome proteins of the chemoautotrophic bacterium *Halothiobacillus neapolitanus* are mostly encoded by genes that are located in a single *cso* operon, including *cbbL* and *cbbS* that encode the large and small subunits of Rubisco, respectively, and genes that encode shell proteins (Fig. 1b). In addition, *csoS1D* is ~11 kbp downstream of the *cso* operon and encodes pesudohexameric shell proteins that are most likely responsible for controlling passage of metabolite molecules^36,37^. We generated the synthetic *cso-1* and *cso-2* operons, which were modified based on the native *H. neapolitanus cso* operon, to heterologously express α-carboxysome shell proteins in *E. coli* BL21(DE3) (Fig. 1b). The *cso-1* operon contains the genes encoding carboxysome shell proteins (*csoS2*, *csoS4AB*, *csoS1CAB*, *csoS1D*), as well as *csoSCA* that encodes β-carbonic anhydrase (CA)^38^. The *cso-2* operon comprises only the shell protein-encoding genes (*csoS2*, *csoS4AB*, *csoS1CAB*, and *csoS1D*), without *csoSCA*. Expression of the *cso-1* and *cso-2* operons both led to production of higher-ordered shell architectures in the *E. coli* hosts, as exhibited by thin-section electron microscopy (EM) (Fig. 1c). Sucrose gradient ultracentrifugation and SDS–polyacrylamide gel electrophoresis (SDS–PAGE) indicated the presence of major shell proteins consisting of CsoS1C, CsoS1A, CsoS1B, and CsoS2 (exists as two isoforms CsoS2A and CsoS2B, see detailed description below) enriched in the 20% sucrose fraction (Fig. 1d). Mass spectrometry further showed the presence of all seven shell proteins and CA encoded by the *cso-1* operon and seven shell proteins encoded by the *cso-2* operon in the 20% sucrose fraction (Supplementary Tables 1 and 2), confirming the self-assembly of expressed carboxysome shell proteins to form shell supramolecular structures.

Negative-staining EM of the 20% sucrose fraction shows that the recombinant α-carboxysome shells exhibit a polyhedral shape, with a diameter of 80–110 nm (*cso-1* shell: 85.93 ± 15.74 nm; *cso-2* shell: 97.12 ± 20.84 nm; *n* = 150) (Fig. 1e), resembling the native α-carboxysomes from *H. neapolitanus*^39^. This shows that empty α-carboxysome shells can be constructed in the non-native host *E. coli*, by expressing only shell proteins without cargo proteins, consistent with in vivo observations^35^. This in turn implies the specific assembly pathway of α-carboxysomes, either ‘shell first’ or ‘concomitant shell–core assembly’, is distinct from the ‘inside–out’ mode of β-carboxysome biogenesis^32,33^.

Without cargos, the shells in the same sucrose fraction exhibit variable structures (Fig. 1e). Likewise, the shell size varies among shell structures collected from different sucrose fractions (Supplementary Fig. 1a, b). Dynamic Light Scattering (DLS) analysis revealed that the shell size gradually increases from 10 to 30% sucrose density (*cso-1* shell: from 82.8 nm to 104.3 nm; *cso-2* shell: from 82.1 nm to 147.8 nm), in agreement with EM results (Supplementary Fig. 1c).

Our results also showed that stable polyhedral shells can be formed in the absence of CA, implicating that CA is not an essential component for shell assembly. This is consistent with previous results, which illustrated that α-carboxysomes can be assembled in *H*. *neapolitanus* without CA^25^. Moreover, the shells lacking CA are on average larger than their counterparts containing CA from individual sucrose fractions (Fig. 1e and Supplementary Fig. 1), suggesting that CA plays a role in defining the shell architecture, likely by tight association with shell proteins^40,41^. The effect of CA on shaping the shell structure is restricted in the shells that possess the minimal size (~82 nm, 10% sucrose, Supplementary Fig. 1b).

### CsoS2 is vital for the formation of empty α-carboxysome shells

**Fig. 2:**
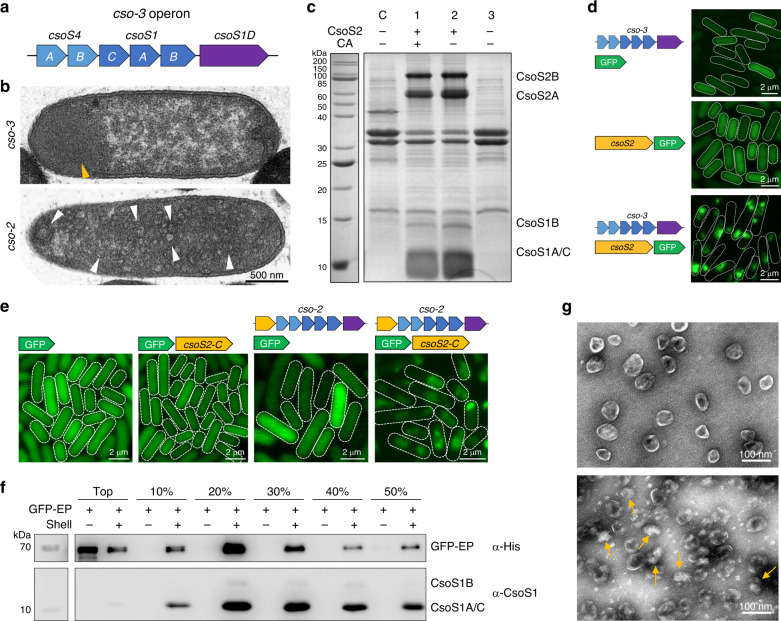
Roles of CsoS2 in carboxysome shell assembly and identification of CsoS2 C-terminus as the encapsulation peptide. **a** Genetic organization of the synthetic *cso-3* operon. **b** Thin-section EM of *E. coli* cells expressing the *cso-3* operon (top) and *cso-2* operon (bottom). Yellow arrows indicate protein aggregates formed by non-assembled shell proteins in the *cso-3* cells. White arrows indicate individual α-carboxysome shell particles in the *cso-2* cells. **c** SDS-PAGE of the 50,000 × *g* pellets purified from *E. coli* cells expressing empty plasmid (control, C), *cso-1* (1), *cso-2* (2) and *cso-3* (3) operon, respectively. **d** Confocal images revealed the formation of shell assemblies in *E. coli* cells in the presence of both CsoS2 and shell proteins. **e** Confocal images of *E. coli* cells expressing GFP, GFP-CsoS2 C-terminus (*csoS2-C*), co-expressing *cso-2* and GFP, or co-expressing *cso-2* and GFP-*csoS2-C*, indicating that the CsoS2 C-terminus could function as an encapsulating peptide to mediate incorporation of external proteins into the formed shell structures. **f** Immunoblot confirms the presence of GFP and shell proteins in the samples purified by sucrose gradient centrifugation from *E. coli* cells producing GFP-EP alone or GFP-EP with shells, using the anti-Histidine (top) and anti-CsoS1A/B/C (below) antibodies (See SDS-PAGE in Supplementary Fig. 3). **g** Transmission EM of empty α-carboxysome shells (top) and shells with GFP interiors (below) from the 20% sucrose fractions. Yellow arrows indicate GFP proteins seen in the lumen of the shells. Source data underlying **b**–**g** are provided as a Source data file.

In the native α-carboxysome, CsoS2 is an intriguing disordered protein and has a relatively high abundance. It exists as two distinct isoforms in *H. neapolitanus*: the longer form CsoS2A (~130 kDa) and the short form CsoS2B (~85 kDa)^42^. CsoS2 interacts with shell proteins and its N-terminus is crucial for organising Rubisco inside the α-carboxysome^43,44^, suggesting the important role of CsoS2 in carboxysome assembly^45^, which is functionally analogous to CcmM, the linking proteins of β-carboxysomes for bridging cargo and the shell^31,46^. To verify the necessity of CsoS2 in the assembly of empty α-carboxysome shells, a *cso-3* operon was generated by deleting *csoS2* from the *cso-2* operon (Fig. 2a). Thin-section EM showed that no shell structures were discerned in the *cso-3 E. coli* cells, in contrast to the EM results of the *cso-2* cells (Fig. 2b). Non-assembled shell proteins were prone to form aggregates at the pole of the *cso-3* cell (Fig. 2b, orange arrow). SDS-PAGE analysis of the pellets of cell extracts after 50,000 × *g* centrifugation showed that shell structures were formed only in the presence of CsoS2 (as indicated by the presence of shell proteins CsoS1A/C and CsoS1B) (Fig. 2c). To further examine the CsoS2-mediated formation of α-carboxysome shells, enhanced green fluorescence protein (GFP) was fused to the C-terminus of full-length CsoS2. Without CsoS2 or shell proteins, GFP signal was evenly distributed throughout the cytosol of *E. coli* (Fig. 2d, top and middle). When CsoS2-GFP and *cso-3* shells were co-expressed, spotty fluorescence signal was visualized in the cell’s cytosol (Fig. 2d, bottom), signifying the formation of highly ordered shell structures. Collectively, our results revealed that CsoS2 is essential for the formation of empty α-carboxysome shells.

### CsoS2 C-terminus acts as the encapsulation peptide to incorporate cargo into the shell

Given the necessity of the intact CsoS2 and its C-terminus in the formation of intact carboxysomes^43–45^ and empty shells (Fig. 2), we speculate that the C-terminal fragment of CsoS2 (278 amino acids) links the shell and cargo assemblies, and thereby, may serve as the EP to direct cargo enzymes into the α-carboxysome shell (Supplementary Fig. 2). To verify this hypothesis, GFP was fused to the N-terminus of the CsoS2 C-terminal fragment and co-expressed with the *cso-2* shells. Confocal images showed that the cells expressing only GFP, GFP-EP, or *cso-2* shells and GFP presented diffuse fluorescence signal throughout the cell, whereas the cells expressing *cso-2* shells and GFP-EP exhibited explicitly dispersed fluorescent foci (Fig. 2e), demonstrating the EP-mediated cargo encapsulation into the synthetic shell. Recombinant *cso-2* shells with incorporated GFP-EP were purified from *E. coli* by sucrose gradient ultracentrifugation. Immunoblot analysis revealed that the shells encapsulating GFP-EP were detected in the 10–50% sucrose fractions, with the strongest GFP signal observed in the 20% sucrose fraction (Fig. 2f and Supplementary Fig. 3). Without shells, by contrast, free GFP-EP were only detected at the top of the sucrose gradient (Fig. 2f), consistent with the quantification of GFP fluorescence in these sucrose fractions (Supplementary Fig. 4). EM of the 20% sucrose fraction revealed the incorporation of GFP-EP in the shell interior, and that the size of the GFP-encapsulated shells is relatively comparable to that of the empty shells (Fig. 2g). These results demonstrate that the C-terminal region of CsoS2 directly interacts with the shell, consistent with the previous finding^43^, and this peptide could serve as an EP to recruit external proteins into the empty shell.

The C-terminal region of CsoS2 has three repeats (R_1_–R_3_) and a conserved C-terminal peptide (Supplementary Fig. 2a)^45^. To determine the roles of these domains in cargo encapsulation, we systematically generated the GFP-tagged CsoS2 C-terminus variants that differ in the number of the repeats and the C-terminal peptide. Coexpressing these GFP-CsoS2 C-terminus peptides with the *cso-2* shells and confocal images showed that the punctate fluorescence signal relative to the cytoplasmic fluorescence reduced along with the decrease in the numbers of the C-terminal repeats (Supplementary Fig. 5). The C-terminal peptide alone was incapable of mediating incorporation of GFP into the shell. These results indicate that the R_1_–R_3_ repeats of the CsoS2 C-terminal region are important for cargo encapsulation, probably by interacting with shell proteins; more repeats ensure higher efficiency of cargo encapsulation. Additionally, removal of the C-terminal peptide from the CsoS2 C-terminus (only R_1_R_2_R_3_) has no significant effects on shell formation and GFP incorporation, supporting the assumption that the C-terminal peptide may be exposed on the outside of the carboxysome shell^43^ (Supplementary Fig. 2d).

### Incorporation of [FeFe]-hydrogenase into the carboxysome shell to create a catalyst for hydrogen production

Though catalytically efficient for hydrogen evolution, [FeFe]-hydrogenases are highly sensitive to O_2_, representing a barrier for biological H_2_ production. The potential O_2_-limited microenvironment in the carboxysome shell is ideal for enhancing the catalytic activities of oxygen-sensitive enzymes. Defining the role of the CsoS2 C-terminus as the EP paves the way for generating a nanoreactor based on the intact α-carboxysome shell, which is capable of incorporating heterologous enzymes for new catalytic functions.

**Fig. 3:**
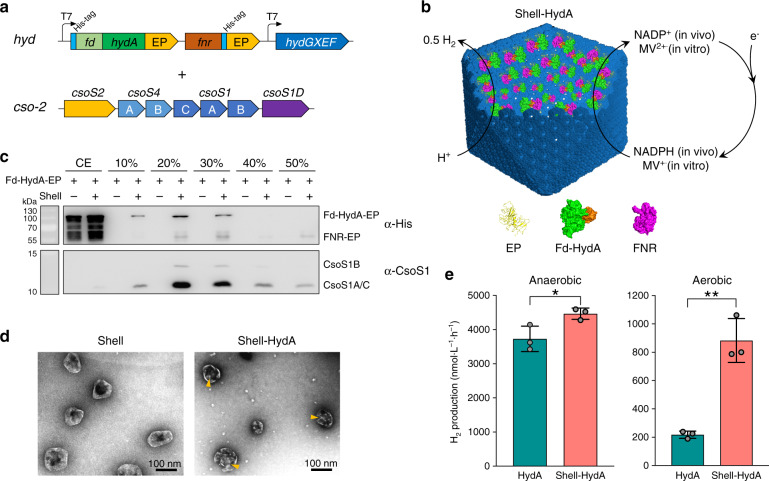
Construction of a hydrogenase-containing nanoreactor based on the carboxysome shell. **a** Generation of the *hyd* operon to produce [FeFe]-hydrogenase HydA fused with ferredoxin (Fd) from the green alga *Chlamydomonas*, ferredoxin:NADP^+^-oxidoreductase (FNR) from *E. coli*, and the HydA maturases HydE, HydF, and HydG. The *hyd* and *cso-2* plasmids were co-expressed to form a hydrogenase-containing nanoreactor based on the carboxysome shell (Shell-HydA). **b** Schematic of the Shell-HydA nanoreactor encapsulating Fd-HydA and FNR. The nanoreactors were tested for hydrogen production activity using endogenous NADPH in cells as the electron donor for in vivo assays, and for in vitro assays, using methyl viologen (MV^+^) as an electron donor, which were chemically reduced by sodium dithionite. **c** Western blot of *E. coli* expressing the *hyd* operon alone or Shell-HydA confirms the presence of Fd-HydA, FNR, and shell proteins in the samples purified from sucrose gradient ultracentrifugation, indicating the assembly of Shell-HydA. **d** Transmission EM of empty shells (left) and Shell-HydA (right). Yellow arrows indicate cargo proteins (Fd-HydA, FNR) in the luminal side of the shell. **e** In vivo hydrogenase activity assays. H_2_ production (nmol L^−1^ h^−1^) of *E. coli* cells expressing free HydA or Shell-HydA grown under anaerobic (left) or aerobic (right) conditions was measured using gas chromatography. Values represent mean ± standard deviations (s.d.), *n* = 3 biologically independent experiments. *p = 0.0352 (left), **p = 0.0018 (right) (two-tailed unpaired *t*-test). Source data underlying **c–e** are provided as a Source data file.

The [FeFe]-hydrogenase from the green alga *Chlamydomonas reinhardtii*, HydA, is one of the simplest [FeFe]-hydrogenases and represents the ‘minimal unit’ for biological H_2_ production^47,48^. We generated a *hyd* plasmid to express the following proteins: (1) algal HydA that are fused with algal Fd at the N-terminus, with a 15-aa linker^49^, and the EP at the C-terminus. Fd serves as the native electron donor of algal HydA, and the Fd-HydA fusion increased the rate of H_2_ production^49^; (2) *E. coli* FNR fused with EP at the C-terminus. FNR originally from *E. coli* could catalyse the transfer of electrons from NADPH to Fd^50^; (3) the maturase enzymes HydE, HydF, and HydG (Fig. 3a), which are crucial for the formation and activation of HydA^51,52^. In addition, polyhistidine tags (His-tags) were linked to the N-terminus of Fd-HydA and the C-terminus of FNR to facilitate biochemical identification. This plasmid and *cso-2* were both transformed into *E. coli* (Fig. 3a). Expression of the *hyd* plasmid was induced by addition of isopropyl β-d-1-thiogalactopyranoside (IPTG) for 4 h before the expression of the shell induced by l-arabinose. This temporally separated expression strategy ensures maturation and activation of hydrogenases prior to shell formation and encapsulation^11^. During shell assembly, EP fusion to both Fd-HydA and FNR directs packaging of Fd, HydA and FNR into the shell interior, allowing for installation of a functional electron transfer pathway into the α-carboxysome shell and generation of a hydrogenase-containing nanoreactor (Shell-HydA) for consecutive H_2_ production using electrons from NADPH (Fig. 3b).

After 50,000×*g* centrifugation, the components of Shell-HydA including shell proteins and HydA were detected in the pellet, whereas unencapsulated HydA were only present in the supernatant (Supplementary Fig. 6), implicating the formation of Shell-HydA assemblies. Similar to the shells containing GFP-EP (Fig. 2f), Shell-HydA were predominantly enriched in the 20% sucrose fraction, whereas expressed HydA in the absence of shells were not detectable in the 10–50% sucrose fractions (Fig. 3c). EM of the Shell-HydA assemblies in the 20% sucrose fraction further suggested the incorporation of HydA into recombinant carboxysome shells mediated by the EP (Fig. 3d, orange arrows).

The H_2_ production activity of the generated *E. coli* cells expressing Shell-HydA and free HydA were assayed using endogenous NADPH in the cells as the electron source. Cells were cultivated and induced for 16 h in either aerobic or anaerobic conditions. The dissolved oxygen (DO) levels of the liquid cultures of two cell types were monitored (Supplementary Fig. 7) and the produced H_2_ was measured by gas chromatography (Fig. 3e). Although both cell types consumed a large amount of O_2_ after 16-h incubation (DO dropped from 25% to 1% for free HydA and from 29% to 1.3% for Shell-HydA), the final DO levels of both cell types under aerobic conditions were notably higher than those kept constant under anaerobic conditions (~0.2%) (Supplementary Fig. 7a). Under anoxic conditions, the H_2_-evolution rate of cells expressing Shell-HydA is 4,464.69 ± 165.49 nmol L^−1^ h^−1^ (mean ± standard deviation (s.d.), *n* = 3), which is ~20% greater than that of cells expressing free HydA (3,729.22 ± 372.00 nmol L^−1^ h^−1^) (Fig. 3e), suggesting that enzyme catalysis can be more efficient in the closed microcompartment likely due to the increased local HydA concentration and more static interaction with substrates. The discrepancy in H_2_ evolution between the *E. coli* hosts producing Shell-HydA and free HydA is explicitly more pronounced under aerobic conditions. The H_2_-evolution rate of cells producing Shell-HydA (882.95 ± 154.71 nmol L^−1^ h^−1^) is ~4.1-fold greater than that of cells expressing free HydA (217.61 ± 25.58 nmol L^−1^ h^−1^) (Fig. 3e). In light of the extreme oxygen sensitivity of [FeFe]-hydrogenases, the significant improvement of the H_2_-producing capacity of Shell-HydA compared to that of free HydA under aerobic condition is mainly ascribed to the lower O_2_ level created within the shell lumen. The possibility that the different hydrogenase activities of the two cell types were ascribed to their distinct oxygen-consumption capacities can be excluded, since no drastic difference in oxygen consumption between the two cell types was determined (Supplementary Fig. 7b). In addition, Shell-HydA cells exhibited a lower H_2_ yield under aerobic conditions than anaerobic conditions, possibly due to the inactivation of O_2_ during the maturation and activation processes of [FeFe]-hydrogenases prior to encapsulation by the shells (Fig. 3e). No notable difference in the H_2_ production activity between HydA encapsulated inside the shell with CA and without CA, under either anaerobic or aerobic conditions was observed (Supplementary Fig. 8).

**Fig. 4:**
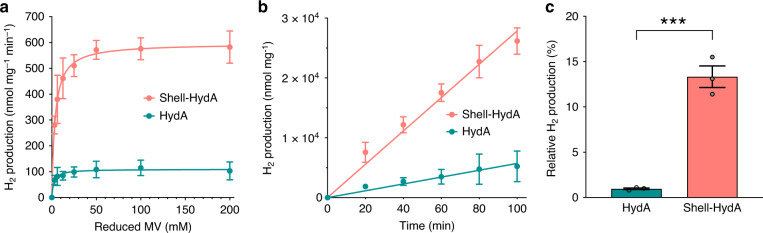
In vitro Hydrogen production of the carboxysome shell-based nanoreactor. **a** Hydrogen production activity (nmol mg^−1^ min^−1^) of isolated free HydA and Shell-HydA at pH 8 as a function of different concentrations of MV^+^ as the electron mediator reduced by sodium dithionite (DT), fitted with Michaelis-Menten kinetics. Values represent mean ± s.d., *n* = 3 biologically independent experiments. **b** Kinetic hydrogen production of free HydA and Shell-HydA using 50 mM DT reduced MV^+^ as the electron mediator at pH 8. Values represent mean ± s.d., *n* = 3 biologically independent experiments. **c** Relative activity of free HydA and Shell-HydA after oxygen exposure for 24 h at 4 °C, as a relative percentage of total activities measured under anaerobic conditions (see also Supplementary Fig. 10). Values represent mean ± s.d., *n* = 3 biologically independent experiments. ***p = 0.0009 (two-tailed unpaired *t*-test). Source data are provided as a Source data file.

The Shell-HydA catalysts were further anaerobically purified for in vitro activity assays using methyl viologen (MV) as an electron donor, which were chemically reduced by sodium dithionite. In the assays, the HydA content was measured using purified HydA as the reference (Supplementary Fig. 9). The maximum hydrogen evolution rate of Shell-HydA is 603.33 ± 44.87 nmol mg^−1^ min^−1^ at pH 8, ~5.5-fold greater than that of free HydA (109.32 ± 29.28 nmol mg^−1^ min^−1^) (Fig. 4a), comparable with the results of in vivo activity assays (Fig. 3e). The amount of hydrogen produced by Shell-HydA measured at 50 mM MV increases linearly over time, indicating that the process is indeed catalytic (Fig. 4b). Moreover, Shell-HydA produces remarkably more hydrogen under these conditions than free HydA, emphasising the advantage of the carboxysome shell-organized nanoreactor in enhancing the H_2_ production activity of encapsulated hydrogenases.

To further investigate the O_2_ tolerance of the shell-based nanoreactor, the Shell-HydA catalysts purified under anaerobic conditions were exposed to the air for 24 h followed by activity assays. After oxygen exposure, Shell-HydA retained 13.33 ± 2.06% activity relative to the activity measured under anaerobic conditions, whereas only 0.97 ± 0.15% activity was maintained for free HydA exposed to O_2_ (Fig. 4c and Supplementary Fig. 10), indicating greater oxygen tolerance of HydA encased inside the carboxysome shell compared to free HydA.

Overall, our results demonstrated that the Shell-HydA nanoreactors are catalytically active for H_2_ production, and encapsulation of the carboxysome shell is beneficial to the H_2_-production activity and oxygen tolerance of encased hydrogenases. Several possibilities could result in the enhancement of catalytic performance of [FeFe]-hydrogenases encapsulated by the carboxysome shell. One possibility is that the selectively permeable shell may create a ‘low-O_2_ microenvironment’ around [FeFe]-hydrogenases by preventing the entry of O_2_^25,53^, thereby promoting the H_2_-production activities of highly oxygen-sensitive [FeFe]-hydrogenases. Shell encapsulation could also allow for condensation of enzymes and more static interaction with substrates within the microcompartment (as indicated by a 20% increase in H_2_ production of Shell-HydA under anaerobic conditions compared to that of free HydA, Fig. 3c), which could facilitate enzyme catalysis^54,55^. Moreover, the protein shell may confer physical protection to its cargo encapsulated from proteolytic cleavage^11^.

It was proposed that the carboxysome shell allows passage of negatively charged metabolites^53,56,57^ and protons^58^ across the shell. The main shell proteins in the α-carboxysome, CsoS1A hexamers, contain a narrow pore with a diameter of ~4 Å^56^, whereas the CsoS1D pseudohexamers have a larger pore with a diameter ranging between 0 and 18 Å^37^. The interior of this channel is mainly positively charged, paving the way for passage of large negatively charged metabolites (such as NADPH) across the shell. The H_2_ production experiments suggested that the positive protons and negative NADPH, as well as the catalytic product H_2_, can diffuse across the shell (Fig. 3b). Precisely how the carboxysome shell permits diffusion of small H_2_ molecules merits further investigation.

In summary, we generated and characterized large and robust carboxysome shells with a diameter of ~100 nm. We also demonstrated the feasibility of reprograming the synthetic protein cages, by sequestering a catalytically active, H_2_ producing pathway within the shell, to boost production of hydrogen. The heterologously engineered shells possess similar protein composition and architectures relative to the carboxysome shells from the native hosts, suggesting the great capacity of cargo loading. We provide evidence that CsoS2 is essential for the assembly of the empty shell, and the C-terminus of CsoS2 could serve as an EP for recruiting external cargo proteins into the synthetic shells. Taking advantage of the defined EP and self-assembly of the carboxysome shells, we installed active [FeFe]-hydrogenases and Fd from green algae together with *E. coli* FNR inside the empty shell to create a large proteinaceous nanoreactor for hydrogen production. One limitation of using endogenous C-terminus of CsoS2 as EP may be the relatively low loading capacity of cargo enzymes, given the limited interaction site of CsoS2 on the shell. Developing efficient encapsulation strategies to improve cargo loading and protein organization is required in future explorations. The shell encapsulation and the specific microenvironment created in the engineering nanoreactor were demonstrated to favour the catalytic activity of oxygen-sensitive [FeFe]-hydrogenases and improve O_2_ tolerance of the generated catalysts significantly. Advanced understanding of the assembly principles of carboxysomes and shells, and the developed engineering systems for precisely tuning enzyme activation, shell formation and encapsulation will inform rational design and engineering of carboxysome shell-based nanomaterials and nanostructures in biotechnological applications for catalytic enhancement, enzyme protection, and molecule delivery. We also see potential to combine these biological assemblies with abiotic cocatalysts in the future.

## Methods

### Generation of constructs

All connections between genes and linearized vectors were achieved by Gibson assembly (Gibson assembly kit, New England BioLabs, UK). The nucleotide sequence of the α-carboxysome shell operon encoding CsoS2, CsoSCA, CsoS4A, CsoS4B, CsoS1C, CsoS1A, CsoS1B, and CsoS1D was amplified from the genome of *Halothiobacillus neapolitanus* and was cloned into the pBAD vector linearized by NcoI and EcoRI (*cso-1* vector). The *csoS2* gene and the nucleotide sequence encoding CsoS4A, CsoS4B, CsoS1C, CsoS1A, CsoS1B, and CsoS1D were amplified from *cso*-1 and assembled into pBAD vector linearized by NcoI and EcoRI (*cso-2* vector). The genes encoding CsoS4A, CsoS4B, CsoS1C, CsoS1A, CsoS1B, and CsoS1D were amplified from the *cso*-1 vector and inserted into pBAD vector linearized by NcoI and EcoRI to generate the *cso-3* vector. The nucleotide sequence of the C-terminus of full-length CsoS2 was amplified from the *cso-1* vector. The *hydA* (GenBank accession code AAL23572.1) and *fd* (GenBank accession code XP_001692808.1) genes from *Chlamydomonas reinhardtii* were codon-optimized for heterologous expression in *E. coli*. The Fd protein was fused to the N-terminus of HydA with a 15 amino acid linker composed of GGGGSGGGGSGGGGS (Supplementary Table 3). The *hydGX and hydEF* genes from *Shewanella oneidensis* (GenBank accession code AE014299.2: 4070148-4074530), which encode the maturases HydE, HydF and HydG were synthesized. The *fnr* gene (GenBank accession code WP_053887749.1) was amplified from the genomic DNA of *E. coli* BL21(DE3). The *fd-hydA* and *fnr* genes were separately fused with the nucleotide sequence of EP and then ligated to pCDFDueT-1 linearized by EcoRI and AscI, together with the *hydGX and hydEF* gene fragments, to generate the *hyd* vector. The *gfp* gene was cloned to pCDFDueT-1 linearized by EcoRI and AscI to generate the pCDF-*gfp* vector. The *gfp* gene with the nucleotide sequence of EP fused at the C-terminus was cloned into pCDFDueT-1 and in frame with the nucleotide sequence encoding 6x polyhistidine tag to create pCDF-*gfp*-EP. The *gfp* gene fused to the C-terminal of full-length *csoS2* gene was inserted into pCDFDueT-1 linearized by EcoRI and AscI to construct pCDF-*csoS2-gfp*. The truncations of the CsoS2 C-terminal region were shown in Supplementary Fig. 5. The genes encoding truncated CsoS2 C-terminals were amplified using PCR from plasmid pCDF-*gfp*-EP. The *gfp* gene fused to the N-terminal of these truncated CsoS2 C-terminal regions were inserted into pCDFDueT-1 linearized by BamHI and AscI. All of these constructs were verified by PCR and DNA sequencing and transformed into *E. coli* DH5α and BL21(DE3) cells.

### Heterogeneously generation of α-carboxysome shells

*E. coli* strains containing the *cso-1*, *cso-2* or *cso-3* vectors were cultivated at 37 °C in Lysogeny Broth (LB) medium containing 100 μg mL^−1^ ampicillin. The expression of these vectors was induced by l-Arabinose (1 mM, final concentration) once the cells reached early log phase (OD_600_ = 0.6). Cells were grown at 25 °C for 16 h with constant shaking and then were harvested by centrifugation at 4000 × *g* for 10 min. The cell pellets were washed with TEMB buffer (5 mM Tris-HCl pH = 8.0, 1 mM EDTA, 10 mM MgCl_2_, 20 mM NaHCO_3_) and resuspended in TEMB buffer supplemented with 10% (v/v) CelLytic B cell Lysis reagent (Sigma-Aldrich) and 1% Protein Inhibitor Cocktail (100x) (Sigma-Aldrich). The cell suspensions were lysed by French Press (Stansted Fluid Power, UK). Cell debris was removed by centrifugation, followed by centrifugation at 50,000 × *g* to enrich α-carboxysome shells. The pellets were resuspended in TEMB buffer and then loaded onto sucrose gradients (10–30% or 10–50%, w/v) followed by ultracentrifugation (BeckMan, XL100K Ultracentrifuge) at 105,000 × *g* for 30 min. Each sucrose fractions were collected and stored at 4 °C.

### Recombinantly expression of GFP, GFP-EP and CsoS2-GFP

*E. coli* strains containing pCDF-*gfp*, pCDF-*gfp*-EP or pCDF-*csoS2-gfp* were grown in LB containing 50 μg⋅mL^−1^ spectinomycin at 37 °C. The expression of these vectors was induced by 0.5 mM (final concentration) isopropyl β-d-thiogalactopyranoside (IPTG) once the cells reached early log phase (OD_600_ = 0.6), followed by cell cultivation for 16 h at 25 °C.

### Expression of mature [FeFe]-hydrogenase and generation of α-carboxysome shells with encapsulated hydrogenases

For the expression of mature, functional [FeFe]-hydrogenases as well as Fd and FNR, *E. coli* cells containing the *hyd* vector grown in LB medium containing 0.2 mM ferric ammonium citrate and 50 μg mL^−1^ spectinomycin was induced by the addition of 0.5 mM IPTG, as well as 0.2 mM l-cysteine and 2.5 mM sodium fumarate (final concentration) at OD_600_ = 0.7–0.8. For the co-expression of α-carboxysome shells and mature hydrogenases to produce Shell-HydA, *E. coli* strains containing the *hyd* vector and the *cso-1* or *cso-2* vector were cultivated in LB medium containing 0.2 mM ferric ammonium citrate, 50 μg mL^−1^ spectinomycin, and 100 μg mL^−1^ ampicillin. The *hyd* expression was induced by the addition of 0.5 mM IPTG at OD_600_ = 0.7. After 4-h induction of the *hyd* expression, the shell expression was induced by 1 mM l-Arabinose, and cells were then grown at 25 °C for 16 h. The *hyd* induction was performed before the expression of shells to allow hydrogenase maturation prior to shell encapsulation. This temporally separated expression strategy ensures maturation and activation of hydrogenases prior to shell formation and encapsulation^11^. The purification of HydA and Shell-HydA was carried out following the method for shell purification mentioned above.

### Hydrogenase activity assay

For in vivo activity assay, *E. coli* strains were first grown aerobically at 37 °C until OD_600_ reached 0.7–0.8. Cells were then transferred to falcon tubes, sealed with rubber turn-over closures (Sigma-Aldrich), and degassed by 100% nitrogen for 15 mins before the addition of IPTG, l-cysteine, and sodium fumarate for anaerobic treatment. For aerobic treatment, culture tubes were sealed without nitrogen degassing process. l-Arabinose was added 4 h after IPTG induction. Then, the cells were grown at 25 °C for 16 h. Hydrogen produced in the culture tubes was detected by gas chromatography. In all, 1 mL gas samples were taken with a gas-tight syringe and a sample loop was flushed (100 µL) with the sample. The sample loop was then switched and run on a Bruker 450-GC gas chromatograph. The system was equipped with a molecular sieve 13 × 60–80 mesh 1.5 m × 1/8 in.  × 2 mm ss column at 50 °C with an argon flow of 40.0 mL min^−1^. Hydrogen was detected by a thermal conductivity detector referencing against standard gas with a known concentration of hydrogen. For each experiment, at least three biological replicates were examined.

For in vitro activity assay, strains were grown aerobically at 37 °C until OD_600_ reached 0.7–0.8, then degassed by 100% nitrogen before the addition of IPTG, L-cysteine and sodium fumarate. l-Arabinose was added 4 h after IPTG induction. Cultures were then cultivated at 25 °C with constant shaking for 16 h. Cell harvesting and protein purification were carried out in an anaerobic chamber (Don Whitley Scientific, MACS-MG-500). The in vitro hydrogen evolution assays were performed inside an anaerobic glove bag (The Versatile AtmosBag, Sigma-Aldrich) flushed with 100% N_2_ before measurements. In vitro activity assay of free HydA was performed as a control for comparison with the hydrogen evolution activity of Shell-HydA.

For in vitro hydrogenase kinetics assays, the protein amount of HydA in the samples containing free HydA or Shell-HydA were quantified by immunoblot using purified HydA as the reference. Then, samples (0.5 mL, 10–15 mg mL^−1^) containing equal amount of HydA in TMB buffer (5 mM Tris-HCl pH 8.0, 10 mM MgCl_2_ and 20 mM NaHCO_3_) were mixed with 100% nitrogen degassed MV (0–200 mM, final) and sodium dithionite (500 mM, final) in serum vials (Agilent Technologies) inside the anaerobic glove bag. The vials were incubated at 37 °C for 100 min with constant shaking and were then assayed for hydrogen production. Hydrogenase activity at a range of MV concentrations was plotted and fit using a standard Michaelis-Menten model. In addition, hydrogen evolution of free HydA and Shell-HydA at 50 mM MV and 500 mM sodium dithionite was measured every 20 min using gas chromatography. For each experiment, at least three biological replicates were examined.

### Oxygen exposure treatment

The HydA and Shell-HydA samples (0.5 mL, 10–15 mg mL^−1^) used in the in vitro activity assays were exposed to the air for 24 h at 4 °C and were then sealed with rubber turn-over closures, followed by 100% nitrogen degassing. The degassed samples were mixed with MV (50 mM, final) and sodium dithionite (500 mM, final). The vials were incubated for 16 h at 37 °C with constant shaking and were then assayed for hydrogen production. All buffers used in the experiments were pre-degassed by 100% nitrogen. For each experiment, at least three biological replicates were examined.

### Dissolved oxygen measurement

The dissolved oxygen (DO) levels of strains expressing HydA or Shell-HydA used for in vivo activity assay were monitored by a polarographic DO probe (New BrunswickTM BioFlo/CellGen 115 Fermentor, Eppendorf) during 16-h induction. The polarized polarographic DO probe was calibrated by oxygen saturated water (100% DO) and sodium dithionite treated water (0% DO), respectively. DO levels of samples were measured before IPTG addition and every four hours after IPTG addition. For each experiment, three biological replicates were examined.

### Mass spectrometry

The shell samples collected from sucrose fractions were washed with PBS buffer and were treated for mass spectrometry analysis^24^. Data-dependent LC−MS/MS analysis was performed on a QExactive quadrupole-Orbitrap mass spectrometer coupled to a Dionex Ultimate 3000 RSLC nano-liquid chromatograph (Thermo Fisher, UK) installed with Xcalibur software version 4.1. A Mascot Generic File, created by Progenesis QI (Version 3.0, Nonlinear Dynamics, Newcastle upon Tyne, UK), was searched against the *H. neapolitanus* carboxysome protein database from UniProt.

### SDS-PAGE and immunoblot analysis

SDS-PAGE and immunoblot examination were performed following the procedure described previously^59^. Briefly, 30 μg of total protein was loaded into each well. Immunoblot analysis was performed using primary mouse monoclonal anti-His (Invitrogen, dilution 1:3000), rabbit polyclonal anti-CsoS1 (Agrisera, dilution 1:3000), and horseradish peroxidase-conjugated goat anti-mouse IgG secondary antibody (Agrisera, dilution 1:10,000) and anti-rabbit IgG secondary antibody (Agrisera, dilution 1:10,000). Signals were visualized using a chemiluminescence kit (Bio-Rad). Immunoblot images were collected by ImageQuant LAS 4000 software version 1.2.1.119. Immunoblot protein quantification was performed using ImageJ software (version 1.52 h). For each experiment, at least three biological repeats were examined.

### Dynamic light scattering analysis

In all, 1.5 mL (20 mg mL^−1^ total protein) of individual sucrose fractions (10%, 15%, 20%, 25%, 30%) containing shell particles were analyzed by Dynamic light scattering (DLS ZetaSizer controlled by malvern zetasizer software version 7.1.1) to measure the size distribution and average size of the shells. For each experiment, at least three biological repeats were examined.

### Transmission electron microscopy

Thin-section transmission electron microscopy (EM) was performed to visualize the reconstituted shell structures in *E. coli* strains^21,59,60^. Isolated shell structures were characterized using negative staining EM^24,29^. Images were recorded using an FEI Tecnai G2 Spirit BioTWIN transmission electron microscope equipped with a Gatan Rio 16 camera. Image analysis was carried out using ImageJ software (version 1.52 h). Statistical analysis was performed using Student’s t-test.

### Confocal microscopy

Overnight induced *E. coli* cells were immobilized by drying a droplet of cell suspension onto LB agar pads as described before^59^. Blocks of agar with the cells absorbed onto the surface were covered with a coverslip and placed under the microscope. Laser-scanning confocal fluorescence microscopy imaging was performed on a Zeiss LSM780 confocal microscope with a 63×/1.4 NA oil-immersion objective with excitation wavelength at 488 nm and emission at 520 nm. Live-cell images were recorded from at least five different cultures. All images were captured with all pixels below saturation. Image analysis was carried out using ImageJ software (version 1.52 h).

### Statistics and reproducibility

All experiments reported here were performed at least three times independently and at least three biological repeats were performed for each experiment.

### Reporting summary

Further information on research design is available in the Nature Research Reporting Summary linked to this article.

## Supplementary information

Supplementary Information

Reporting summary

## Data Availability

Data supporting the findings of this work are available within the paper and its Supplementary Information files. A reporting summary for this article is available as a Supplementary Information file. All data are available from the corresponding author upon request. The protein sequence of Fd, HydA and FNR are available in NCBI with the accession code XP_001692808.1, AAL23572.1 and WP_053887749.1, respectively. The protein sequence of HydGXEF is available in NCBI with the accession code AE014299.2 (region 4070148-4074530). The protein sequence in Supplementary Fig. 2b is available in NCBI with the accession code WP_081441107.1. Source data are provided with this paper.

## Source data

Source data
