# Supplementary material for: Reprogramming bacterial protein organelles as a nanoreactor for hydrogen production
Source: Nat Commun. 2020 Oct 28;11:5448. doi: 10.1038/s41467-020-19280-0 (PMC7595155; doi:10.1038/s41467-020-19280-0)
Supplement: Supplementary file 1 — Supplementary Information [file 41467_2020_19280_MOESM1_ESM.pdf]

Reprogramming bacterial protein organelles as a nanoreactor for hydrogen production

Li et al.

Supplementary Table 1. Mass spectroscopic identification of α-carboxysome proteins (shell proteins + CA) expressed and isolated from the pBAD-*cso1 E. coli* construct. Source data of Supplementary Table 1 are provided as a Source Data file.

Protein	UniProt	MW (kDa)	Mascot score	Coverage	Selected unique peptides
CsoS2	D0KZ90	91.3	10155.22	67.21%	AAQAPVVQPEVVATQEKPELVcAPR<br>AVTGTEYVGTEQFTSFcNTSPKPNATK<br>ELVGSPQPMAMAMANR<br>FNQTGNVQSMGFK<br>GQSVTGNLVDRSELVTGNEPGScSR<br>GTSVSGQQLDHAPK<br>ITGSSGNDTQGSLITYSGGAR<br>KSHVTGNEPGTCR<br>KVETGHTLSGGSVTGTQVDR<br>NGYQIDGYLDTALHGR<br>NTNQPEQNFAPGEVMPTDFSIQTPAR<br>NVTGTEYMSNEAHFSLcGTAAKPSQADK<br>SELVTGNEPGSCSR<br>SISGTEYLSNEQFQSFcDTKPQR<br>SSVNEVPTDYQMADQLcSTIDHADFGTESNR<br>THQVVSGSDEFRPSSVTGNESGAK<br>TNKGQSVTGNLVDR<br>VETGHTLSGGSVTGTQVDR<br>VRPQQSVVHNDAmIENAGDTNQSSSTSLNNELSEI<br>CSIADDMPER
CsoS1A	P45689	10.0	2959.46	78.57%	ADVTGIALGMIETR<br>MADVTGIALGMIETR
CsoS1C	P45688	9.9	2739.59	77.55%	AAVTGIALGMIETR
CsoS1B	P45690	11.3	2092.56	59.09%	ATTHGIALGMIETR<br>SFVGGGYVTVMVR
CsoSCA	D0KZ89	57.3	648	35.99%	ALTAATEQFSR<br>APFGVSSSVKPR<br>DNWISGHNLK<br>FSSLDEQNLLQFR<br>GLPIPIAIHYR<br>GNGVASEGMR<br>HPLTDLEANEQLGR<br>LDLIEQAPNPAYDR<br>VIIHEAILNANR<br>VQELAQSSLGHTLPEELLK<br>YISVGDGFDEVQIR<br>YVDNTVTYAQTLHLAPDEAR
CsoS1D	D0KZ73	23.4	538.33	48.36%	AAEATIASINAR<br>AAHVTLIDVR<br>AFGSMEIHYR<br>AITPDHATLINR<br>GSMLLPGK<br>LGEQVVER<br>LSDIALK<br>LTMMGSEAETEEAMR<br>MNNIDLR<br>NQSDVLASGEAVLR
CsoS4B	D0KZ87	8.8	86.13	39.51%	SDLIATR<br>VSVAcDPIGVPEGcWVFTISGSAAR
CsoS4A	D0KZ88	8.9	69.91	37.35%	QVAVDAIGcIPGDWVLcVGSSAAR<br>TLVSTNR

Supplementary Table 2. Mass spectroscopic identification of α-carboxysome shell proteins expressed and isolated from the pBAD-*cso2 E. coli* construct. Source data of Supplementary Table 2 are provided as a Source Data file.

Protein	UniProt	MW (kDa)	Mascot score	Coverage	Unique peptides
CsoS2	D0KZ90	91.3	10173.05	69.64%	AAQAPVVQPEVVATQEKPELVcAPR<br>AVTGTEYVGTEQFTSFcNTSPKPNATK<br>ELVGSPQPMAMAMANR<br>FNQTGNVQSMGFK<br>GQSVTGNLVDRSELVTGNEPGScSR<br>GTSVSGQQLDHAPK<br>ITGSSGNDTQGSLITYSGGAR<br>KSHVTGNEPGTCR<br>KVETGHTLSGGSVTGTQVDR<br>NGYQIDGYLDTALHGR<br>NTNQPEQNFAPGEVMPTDFSIQTPAR<br>NVTGTEYMSNEAHFSLcGTAAKPSQADK<br>SELVTGNEPGSCSR<br>SISGTEYLSNEQFQSFcDTKPQR<br>SSVNEVPTDYQMADQLcSTIDHADFGTESNR<br>THQVVSGSDEFRPSSVTGNESGAK<br>TNKGQSVTGNLVDR<br>VETGHTLSGGSVTGTQVDR<br>VRPQQSVVHNDAmIENAGDTNQSSSTSLNNELSEI<br>CSIADDMPER
CsoS1A	P45689	10.0	3822.30	78.57%	ADVTGIALGMIETR
CsoS1C	P45688	9.9	3763.76	77.55%	AAVTGIALGMIETR
CsoS1B	P45690	11.3	2580.58	84.55%	ATTHGIALGMIETR<br>MATTHGIALGMIETR<br>SFVGGGYVTVMVR<br>VHSEVEIILPETPEDSDSAWCIANLNS
CsoS1D	D0KZ73	23.4	642.85	48.83%	AAEATIASINAR<br>AAHVTLIDVR<br>AFGSMEIHYR<br>AITPDHATLINR<br>GSMLLPGK<br>LGEQVVER<br>LSDIALK<br>LTMMGSEAETEEAMR<br>MNNIDLR<br>NQSDVLASGEAVLR
CsoS4B	D0KZ87	8.8	57.30	37.35%	SDLIATR<br>VSVACDPIGVPEGcWVFTISGSAAR
CsoS4A	D0KZ88	8.9	55.51	8.64%	QVAVDAIGCIPGDWVLCVGSSAAR<br>TLVSTNR

Supplementary Table 3. Primers used in this study (overlap sequences for Gibson Assembly are underlined)

Primer	Nucleotide sequence	Comment
pBAD-CsoS2-FW	GGCTAACAGGAGGAATTAACCATGGGGTCAAACATGCCTTC	construction of *cso*-1 , *cso*-2, *cso*-3 operon
CsoS1D-FW	CTAGTAGGGAAGATGCGCATGAACAAC	
CsoS1B-RV	CATGCGCATCTTCCCTACTAGACATTAGCTATTCAGATTTGCGATACACC	
pBAD-CsoS1D-RV	TGTTCTACGTAAGCTTCGTTATTAGAACCCTTCAGCGCGAC	
CsoS4A-CsoS2-RV	CTGCGCCTGAACGCTCAAAGATCGTTACACTTTACTTAATCAACCG	
RBS-CsoS4-FW	TTTGAGCGTTCAGGCGCAG	
pBAD-RBS-CsoS4A-FW	GGCTAACAGGAGGAATTAACTTTGAGCGTTCAGGCGCAG	
pBAD-seq-FW	ATTTGCACGGCGTCACACTT	sequencing primers
pBAD-seq-RV	TCATCCGCCAAAACAGCCAA	
CsoS1C seq-FW	GGTGGTTACGTGACCGTT	
CsoS2-seq-FW	GATCTGTTACGGGCACGCAA	
pCDF-GFP-FW	ATCACCACAGCCAGGATCCGGTGAGCAAGGGCGAGGAGCTGTT	Construction of pCDF-GFP
pCDF-GFP-RV	CAAGCTTGTCGACCTGCAGGTTACTTGTACAGCTCGTCCATGCCG	
Overlap-GFP-FW	CACCATGGTAGCGGTAGTGGAGTGAGCAAGGGCGAGGAGCTGTT	Construction of pCDF-GFP-EP; pCDF-CsoS2-GFP
CsoS2-Cterm-GFP-RV	TCCACTACCGCTACCATGGTGCTTGTACAGCTCGTCCATGCCG	
pCDF-CsoS2-Cterm-RV	CAAGCTTGTCGACCTGCAGGGATCGTTACACTTTACTTAATCAACCG	
pCDF-CsoS2-FW	ATCACCACAGCCAGGATCCGATGGGGTCAAACATGCCTTC	
Overlap-CsoS2-RV	TCCACTACCGCTACCATGGTGACCGCGCGCGCCGCCGGAGTAA	
EGFP-*S2C*-Rv	CTTGTACAGCTCGTCCATGC	construction of pCDF-GFP-*csoS2C ΔR1;* pCDF-GFP-*csoS2C ΔR1,2;* pCDF-GFP-*csoS2C ΔR1,2,3* and pCDF-GFP-*csoS2C ΔC90*
pCDF-S2-RV	CAAGCTTGTCGACCTGCAGGTCAACCGCGCGCGCCGCCGGAGT	
GFP-*S2CΔR1*-Fw	GCATGGACGAGCTGTACAAGCTTCCCACTAGTCCACGCTT	
GFP-*S2CΔR1,2*-Fw	GCATGGACGAGCTGTACAAGAGGCACGCTGCGCGCGAGT	
GFP-*S2CΔR1,2,3*-Fw	GCATGGACGAGCTGTACAAGGCGAGTGGTCGCAACCCTTC	
S2C-ΔC90(pCD)-Rv	GCAGCGGTTTCTTTACCAGACTTATTTGGCACATTGCGATTGG	
pCD (*S2C-ΔC90*)-Fw	GTCTGGTAAAGAAACCGCTGC	
pCDF-seq-FW	CCTTATGCGACTCCTGCAT	sequencing primers
pCDF-MCS1-seq-RV	GGCCGTGTACAATACGATTAC	
pCDF-MCS2-seq-RV	GCTAGTTATTGCTCAGCGG	
pCDF-Fd-HdyA-FW	ATCACCACAGCCAGGATCCGGGTACCGCCTATAAAGTTACCCTGA	construction of the *hyd* plasmid
Fd-HdyA-RV	AGGCCAGTCTTGTGCTCCGA	
hydA-CsoS2-Cterm-FW	TCGGAGCACAAGACTGGCCTCCGTTTTGTACGAGCACCCCAGAG	
FNR-CsoS2-Cterm-RV	CAGCCATACCTGTTCCTCTGTGGATCGTTACACTTTACTTAATCAACCG	
RBS-FNR-FW	CACAGAGGAACAGGTATGGCTGATTGGGTAACAGGC	
His-FNR-RV	TCCACTACCGCTACCATGGTGGTGATGATGGTGCCAGTAATGCTCCGCTGTCA	
His-CsoS2-Cterm-FW	CACCATGGTAGCGGTAGTGGACCGTTTTGTACGAGCACCCCAGAG	
pCDF-hydGX-FW	CATATGGCAGATCTCAATTGGATAAGCACACACGAGCATCACTC	
Hyd GX-RV	TAAATTCCAACTCAGTGGCGAGCT	
hydEF-FW	AGCTCGCCACTGAGTTGG	
pCDF-hydEF-RV	GCAGCGGTTTCTTTACCAGACTTACTATTGCTGAGGATTGCGGT	
hydA-seq-FW	TCATAAAGCACATGAGCTGC	sequencing primers
hydGX-seq-FW	GGAATTGACGATGTCGGCAT	
hydEF-seq-FW	GCAAGAGAGCAGGCACTCA	

linker	Nucleotide sequence	Amino acid sequence
Fd-HydA linker (15 amino acids)	GGAGGTGGTGGTAGCGGTGGTGGTGGTTCAGGTGGTGGCGGTAGC	GGGGSGGGGSGGGGS

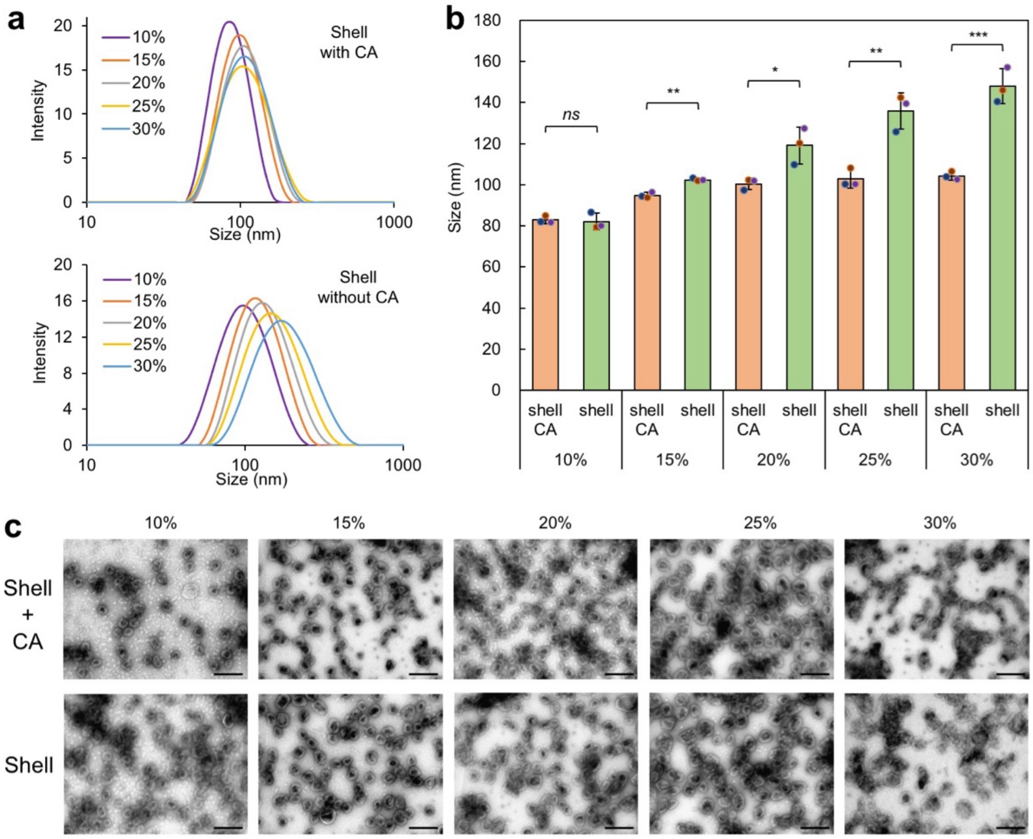

Supplementary Fig. 1. Comparison of the synthetic α-carboxysome shells and shells with CA

a Diameters of α-carboxysome shells with CA and shells in each sucrose fractions (from 15% to 30%) after sucrose gradient centrifugation measured by Dynamic Light Scattering (DLS). **b** Average diameters of shells with CA and shells in each sucrose fractions measured by DLS from three biological repeats. Values represent mean ± s.d., $n = 3$ biologically independent experiments. *ns*, non-significant difference,* $p < 0.05$, ** $p < 0.01$, *** $p < 0.001$ ($p1 = 0.00125$, $p2 = 0.02620$, $p3 = 0.00460$, $p4 = 0.00098$, two-tailed unpaired *t*-test). **c** TEM micrographs of shells with CA and shells in each sucrose fraction. Scale bar: 200 nm. Source data of Supplementary Fig. 1a–c are provided as a Source Data file.

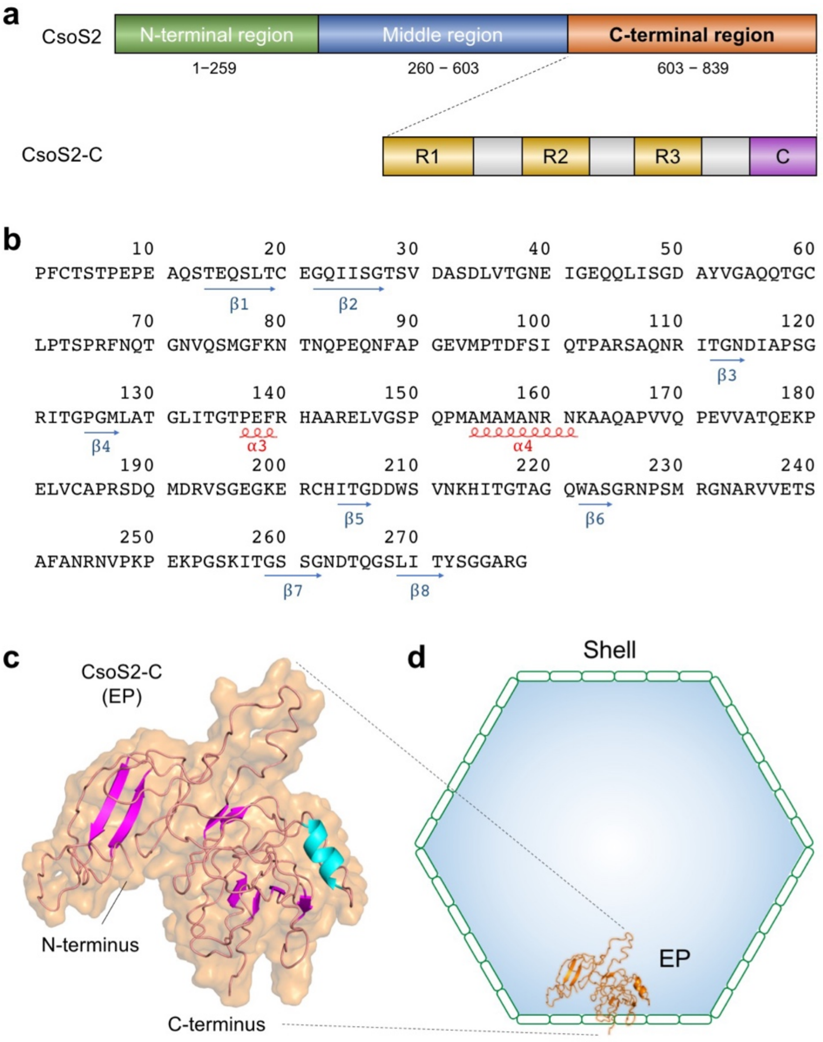

Supplementary Fig. 2. Analysis of CsoS2, CsoS2 C-terminal region and its possible location inside the shell

a A schematic representation of CsoS2, including the N-terminal region, middle region, and C-terminal region. The C-terminal region has three repeats (R_1–R_3) and a conserved C-terminal peptide. **b** The amino acid sequence of the CsoS2 C-terminal region as an EP. The secondary structure formed by specific peptides was denoted below the sequence, analysed by ESPript 3.0. **c** Ribbon presentation of the EP structure predicted by I-TASSER. **d** A model for the location of EP in the luminal side of the shell. The C-terminal peptide (603-839 aa) may be exposed on the outside of the carboxysome shell.

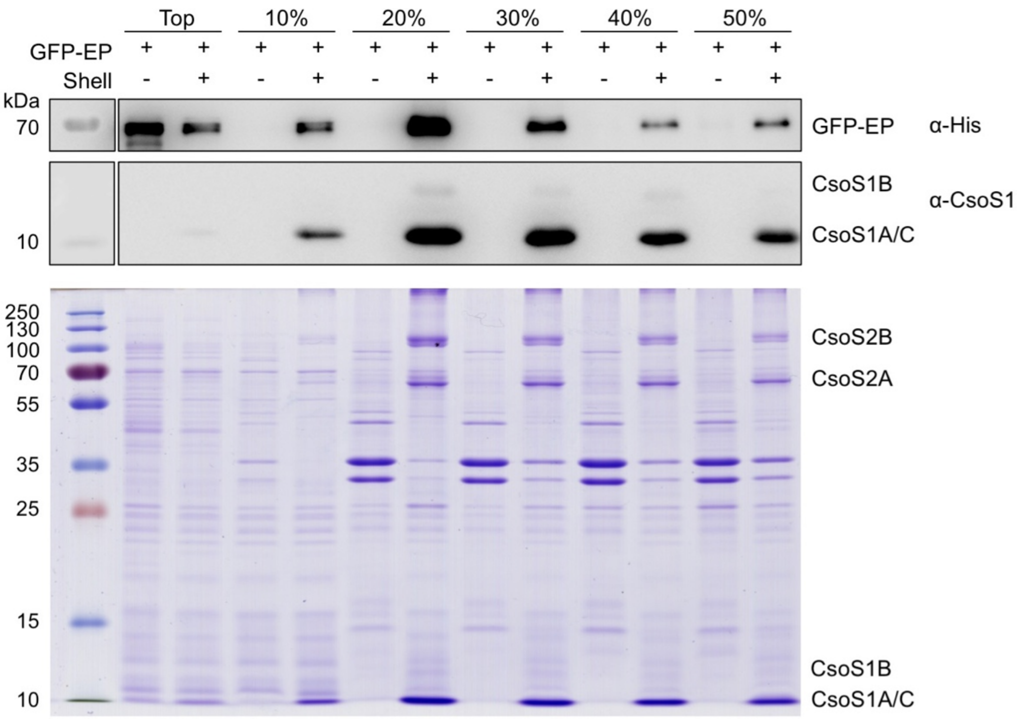

Supplementary Fig. 3. Unencapsulated GFP-EP was not present in the 10%-50% sucrose fractions Immunoblot analysis and SDS-PAGE of recombinant proteins from strains producing GFP-EP alone or co-expressing GFP-EP with shells in each sucrose fractions from 10% to 50%, as well as the top loading layer (Top). Immunoblotting was performed using anti-His antibody (upper panel) and CsoS1 antibody (middle panel), respectively. SDS–PAGE was stained with Coomassie brilliant blue (bottom panel). 30 µg of total protein was loaded on each lane. Source data of Supplementary Fig. 3 are provided as a Source Data file.

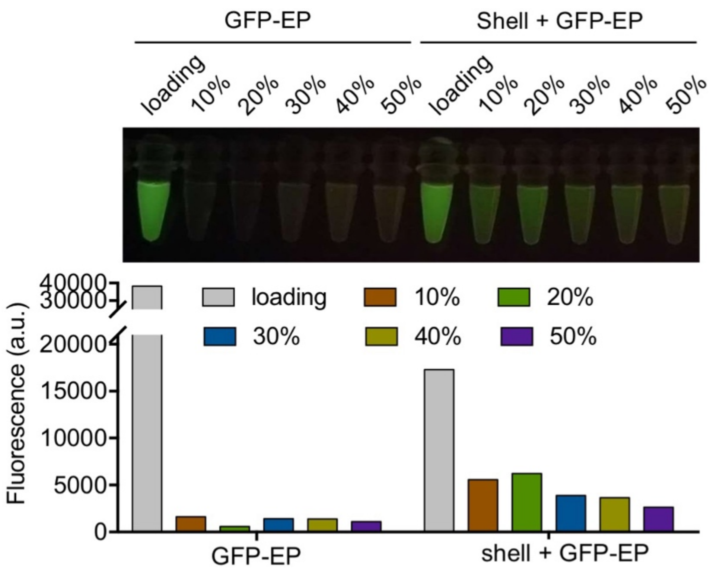

Supplementary Fig. 4. Quantification of GFP fluorescence in each sucrose fractions

GFP fluorescence imaging of recombinant proteins (150 μL in each PCR reaction tube) purified by sucrose gradient centrifugation from strains producing GFP−EP alone or co-expressing GFP with shells shown under 480 nm LED illumination (upper panel). Quantification of the GFP fluorescence (Emission at 520 nm) of the same protein samples for excitation at 488 nm measured by microplate reader (lower panel). Source data of Supplementary Fig. 4 are provided as a Source Data file.

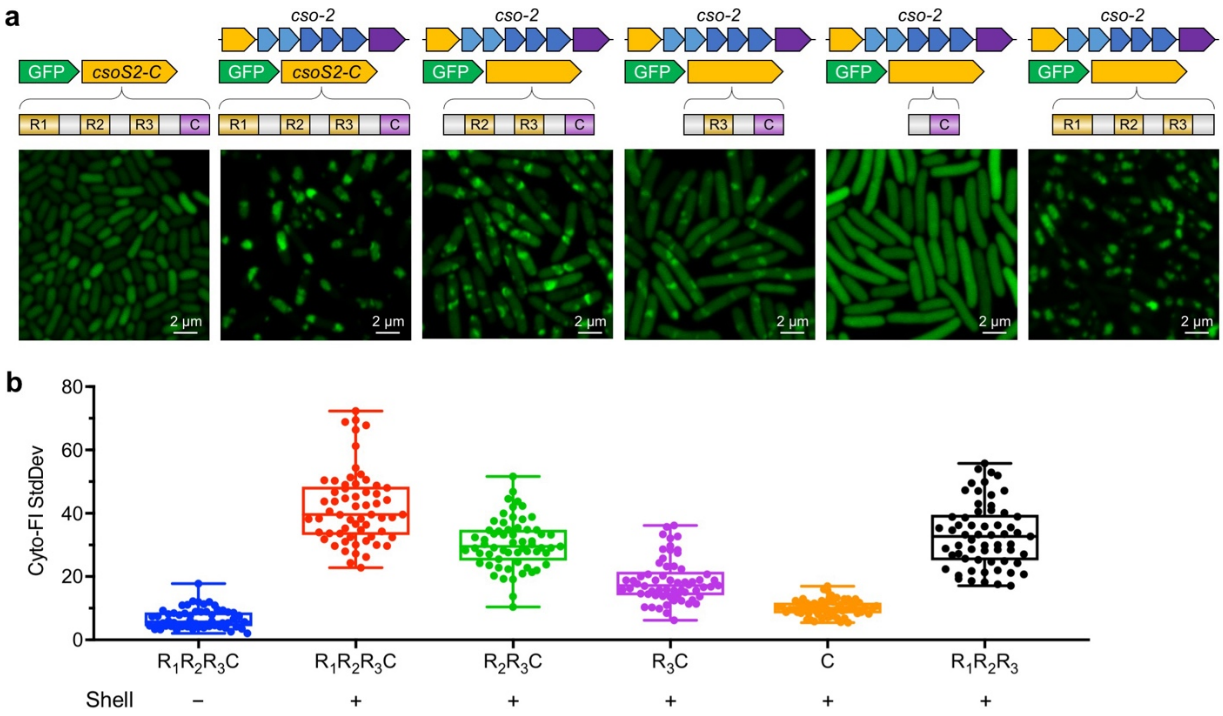

Supplementary Fig. 5. Effects of the three repeats (R_1–R_3) and the C-terminal peptide (C) of the CsoS2 C-terminus on cargo encapsulation.

a Truncations of the GFP-CsoS2 C-terminus and confocal images showing the effects of the R_1–R_3 and C domains of the CsoS2 C-terminus on cargo encapsulation. **b** Analysis of the cytoplasm fluorescence standard deviation showing the variable encapsulation efficiencies of different truncated GFP-csoS2-C proteins. Box and whisker plots indicate the median (middle line in the box), 25th percentile (bottom line of the box), 75th percentile (top line of the box), as well as the minima and maxima (whiskers) (n = 60). Source data of Supplementary Fig. 5a-b are provided as a Source Data file.

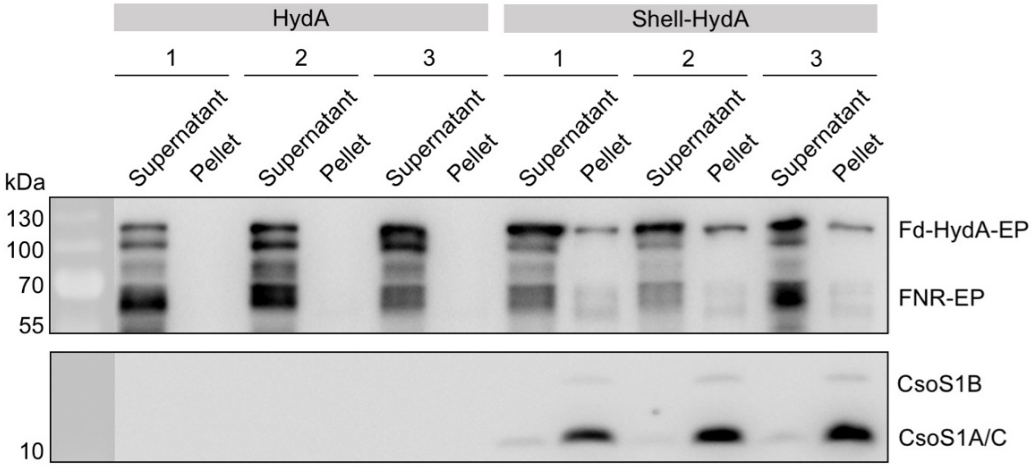

Supplementary Fig. 6. Unencapsulated HydA only existed in the supernatant of cell extracts after 50,000 *g* centrifugation

Immunoblot analysis of the supernatants and pellets of cell extracts after 50,000 *g* centrifugation from strains producing HydA alone or co-expressing HydA with α-carboxysome shells (including three biological repeats) using 6×His antibody (upper panel) and CsoS1 antibody (lower panel), respectively. 30 μg of total protein was loaded on each lane. Source data of Supplementary Fig. 6 are provided as a Source Data file.

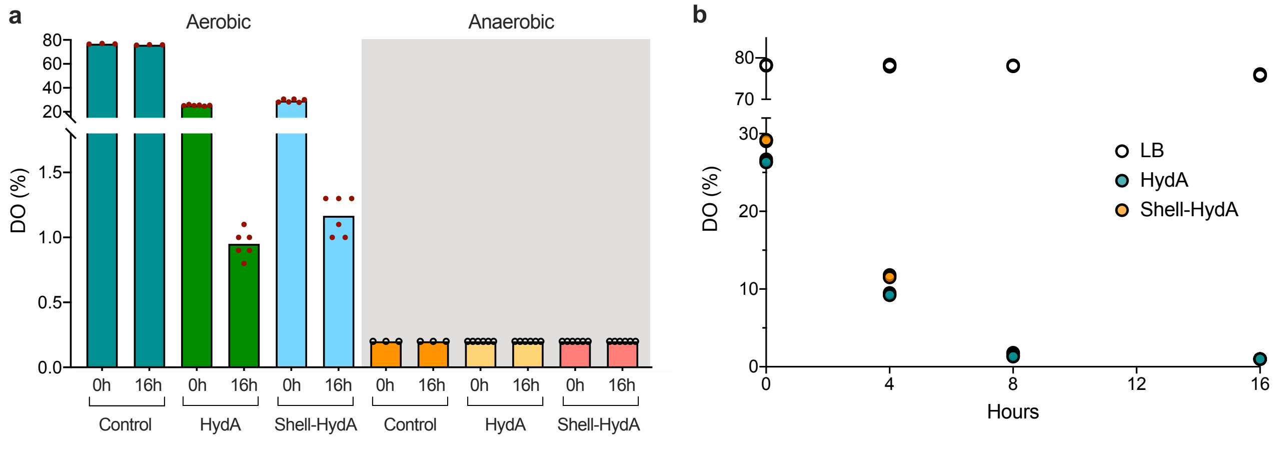

Supplementary Fig. 7. No significant difference in oxygen consumption of cells expressing HydA or Shell−HydA under aerobic condition and anaerobic condition during 16-hour induction.

a The initial and final dissolved oxygen (DO) in LB medium (control), LB medium containing live cells producing HydA or Shell-HydA under either aerobic or anaerobic condition during 16-hour induction. Water saturated DO was set as 100%. The data are presented as the average of six (or three for control) DO measurements of six (or three) distinct cell cultures. **b** DO changes in the LB medium (black line), cells producing HydA (teal) or Shell−HydA (orange) under aerobic conditions during 16-hour incubation. The data are presented as three DO measurements of three distinct cell cultures. Source data of Supplementary Fig. 7a−b are provided as a Source Data file.

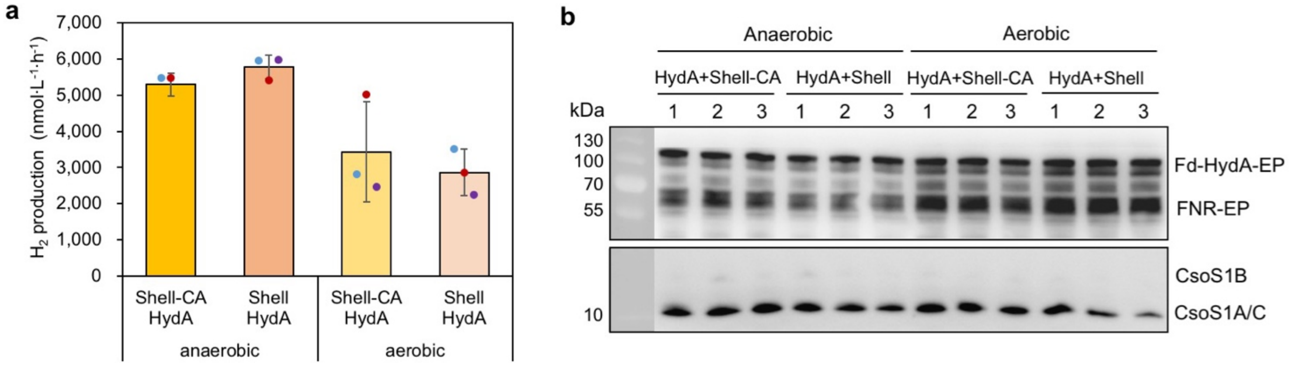

Supplementary Fig. 8. No significant difference in hydrogen productivity between HydA encapsulated inside the shell with and without CA

a *In vivo* hydrogen evolution assays of cell cultures expressing HydA and shells with/without CA under either anaerobic or aerobic condition after 16-hour incubation were conducted using gas chromatography. Values represent mean ± s.d., n = 3 biologically independent experiments. **b** Immunoblot analysis of whole cell extracts of the same samples (including three biological repeats). 6×His antibody was used for Fd-HydA and FNR detection (upper panel). Shell proteins CsoS1A/B/C were detected by CsoS1 antibody (lower panel). Source data of Supplementary Fig. 8a−b are provided as a Source Data file.

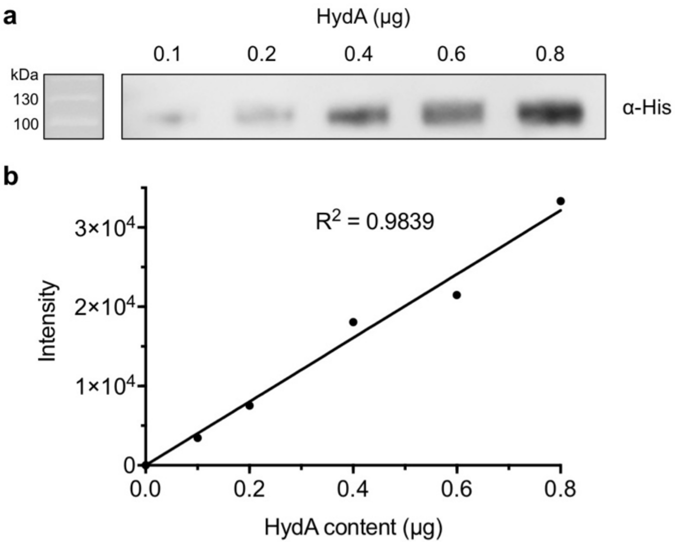

Supplementary Fig. 9. A linear correlation was observed between quantitation of purified HydA by BCA protein assay kit and densitometric quantitation from immunoblot

a Immunoblot analysis of purified HydA (Fd-HydA-EP) using 6×His antibody. HydA was purified by immobilized-nickel affinity chromatography followed by quantification by BCA protein assay kit. 0.1 µg, 0.2 µg, 0.4 µg, 0.6 µg and 0.8 µg of HydA was loaded onto each well of the gel, respectively. **b** Linear relationship between HydA content quantified by BCA protein assay kit and the corresponding HydA content quantification by immunoblot. Densitometric quantitation of HydA levels were determined using ImageJ. Source data of Supplementary Fig. 9a−b are provided as a Source Data file.

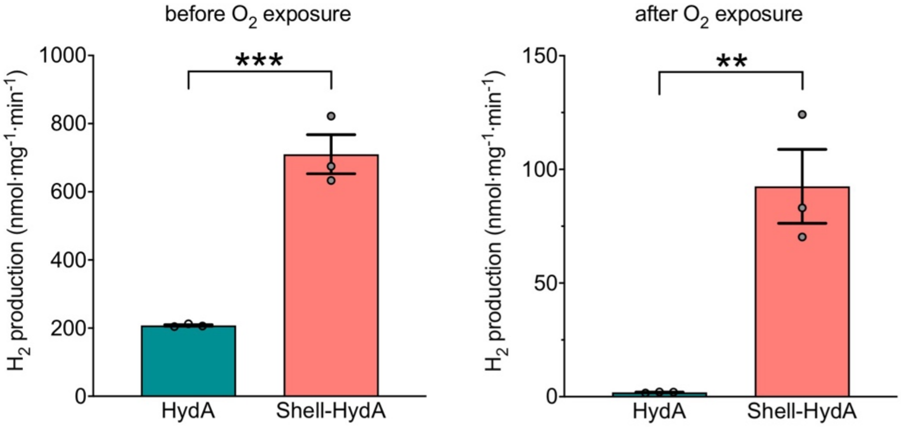

Supplementary Fig. 10. H_2-production activity of free HydA and Shell-HydA before (left) and after (right) oxygen exposure for 24 hours at 4 °C (see also Fig. 4c).

Values represent mean ± s.d., $n = 3$ biologically independent experiments. *** $p = 0.0009$ (left), **$p = 0.0051$ (right) (two-tailed unpaired t-test). Source data of Supplementary Fig. 10 are provided as a Source Data file.
