# Supplementary figures and images for: Reprogramming bacterial protein organelles as a nanoreactor for hydrogen production
Source: Nat Commun. 2020 Oct 28;11:5448. doi: 10.1038/s41467-020-19280-0 (PMC7595155; doi:10.1038/s41467-020-19280-0)

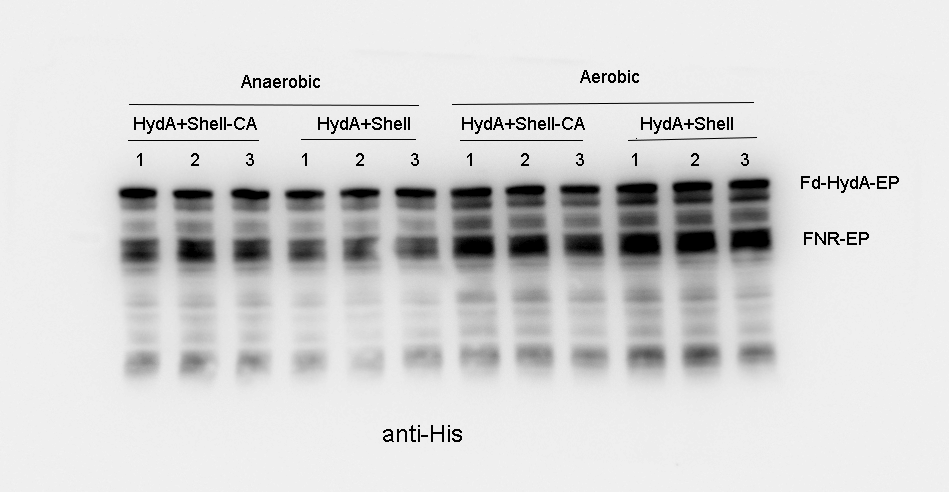

Supplement: Supplementary file 3 — Source data [file 41467_2020_19280_MOESM3_ESM.zip › Source Data_R2/supplementary Fig. 8/supplementary Fig. 8b anti-His.tif]

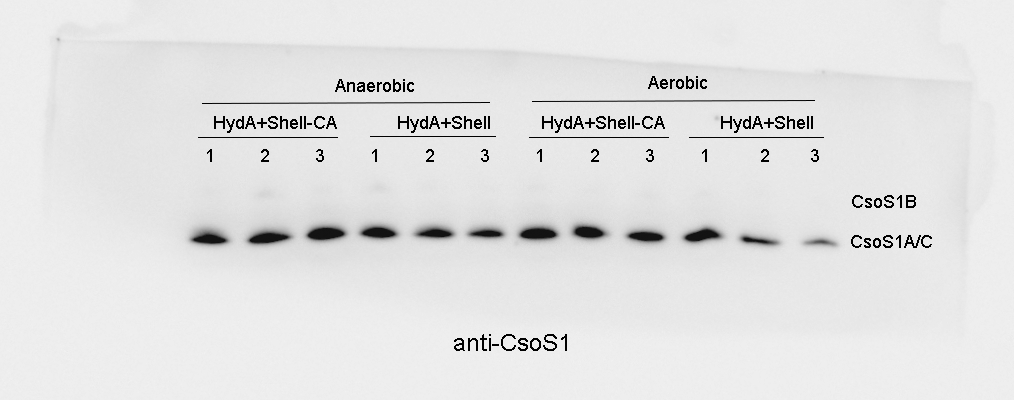

Supplement: Supplementary file 3 — Source data [file 41467_2020_19280_MOESM3_ESM.zip › Source Data_R2/supplementary Fig. 8/supplementary Fig. 8b anti-CsoS1.tif]

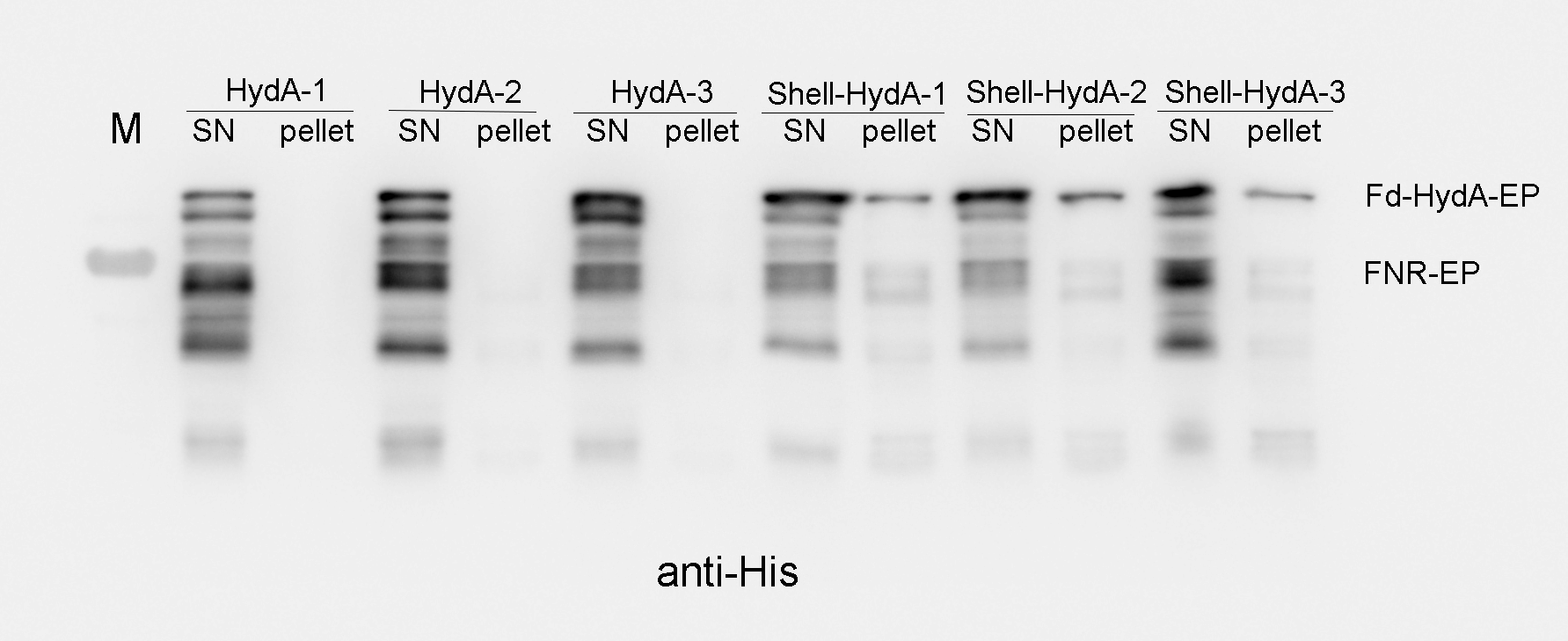

Supplement: Supplementary file 3 — Source data [file 41467_2020_19280_MOESM3_ESM.zip › Source Data_R2/supplementary Fig. 6/anti-His.tif]

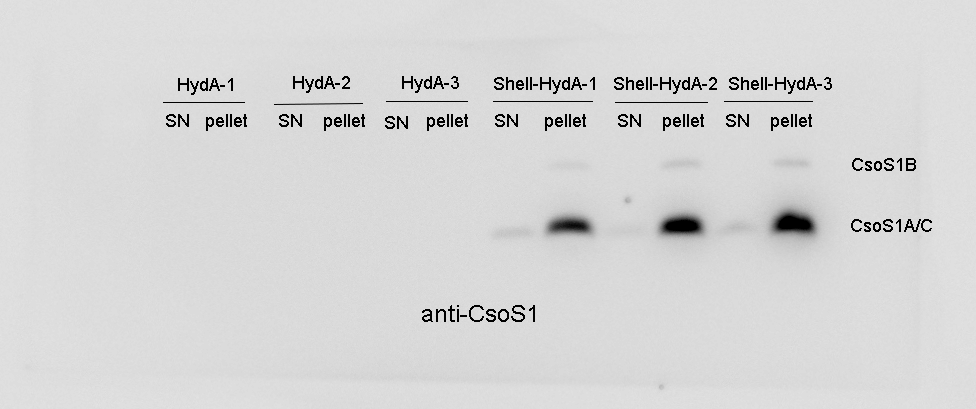

Supplement: Supplementary file 3 — Source data [file 41467_2020_19280_MOESM3_ESM.zip › Source Data_R2/supplementary Fig. 6/anti-CsoS1.tif]

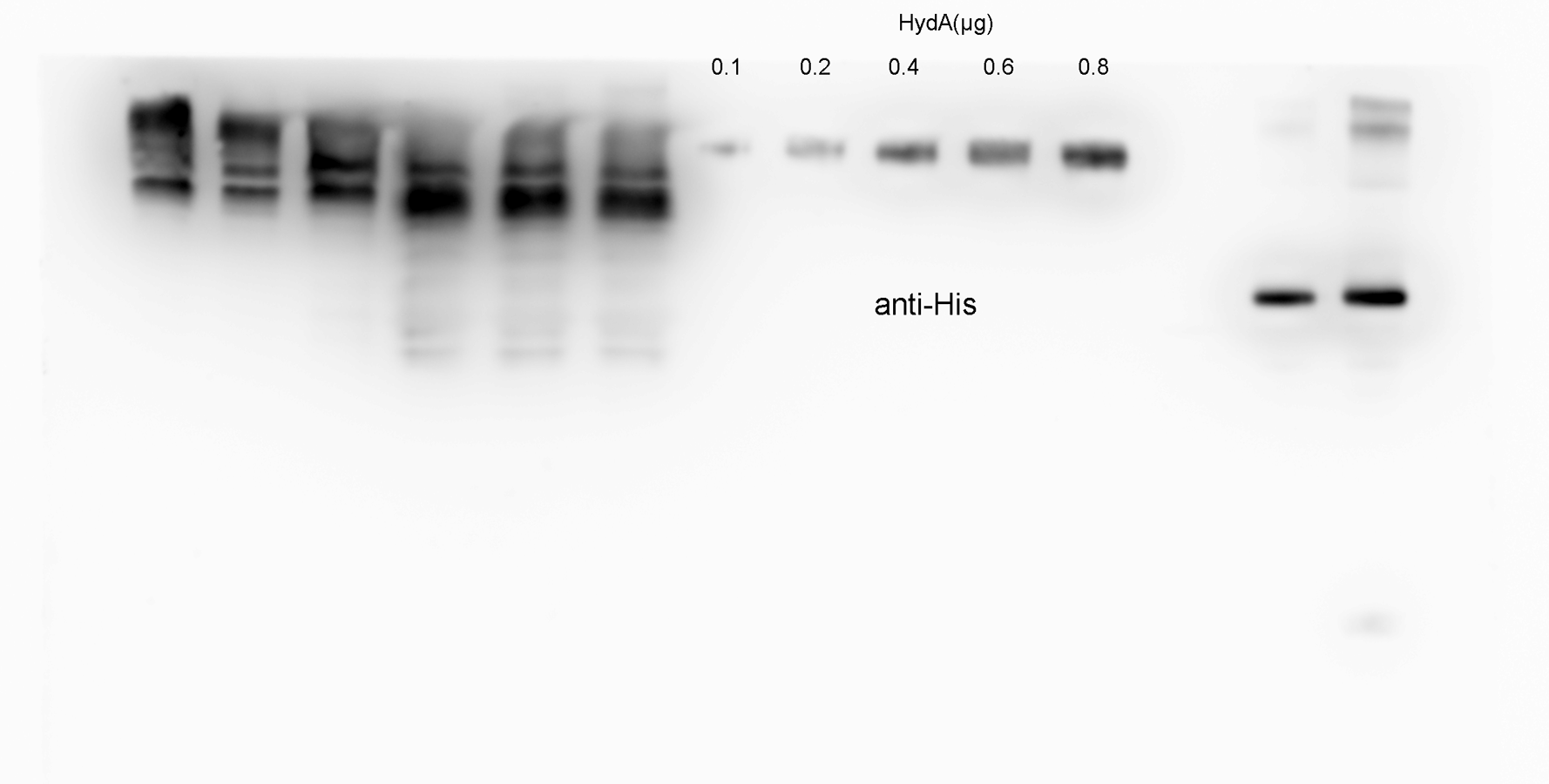

Supplement: Supplementary file 3 — Source data [file 41467_2020_19280_MOESM3_ESM.zip › Source Data_R2/supplementary Fig. 9/suplementary Fig 9a.tif]

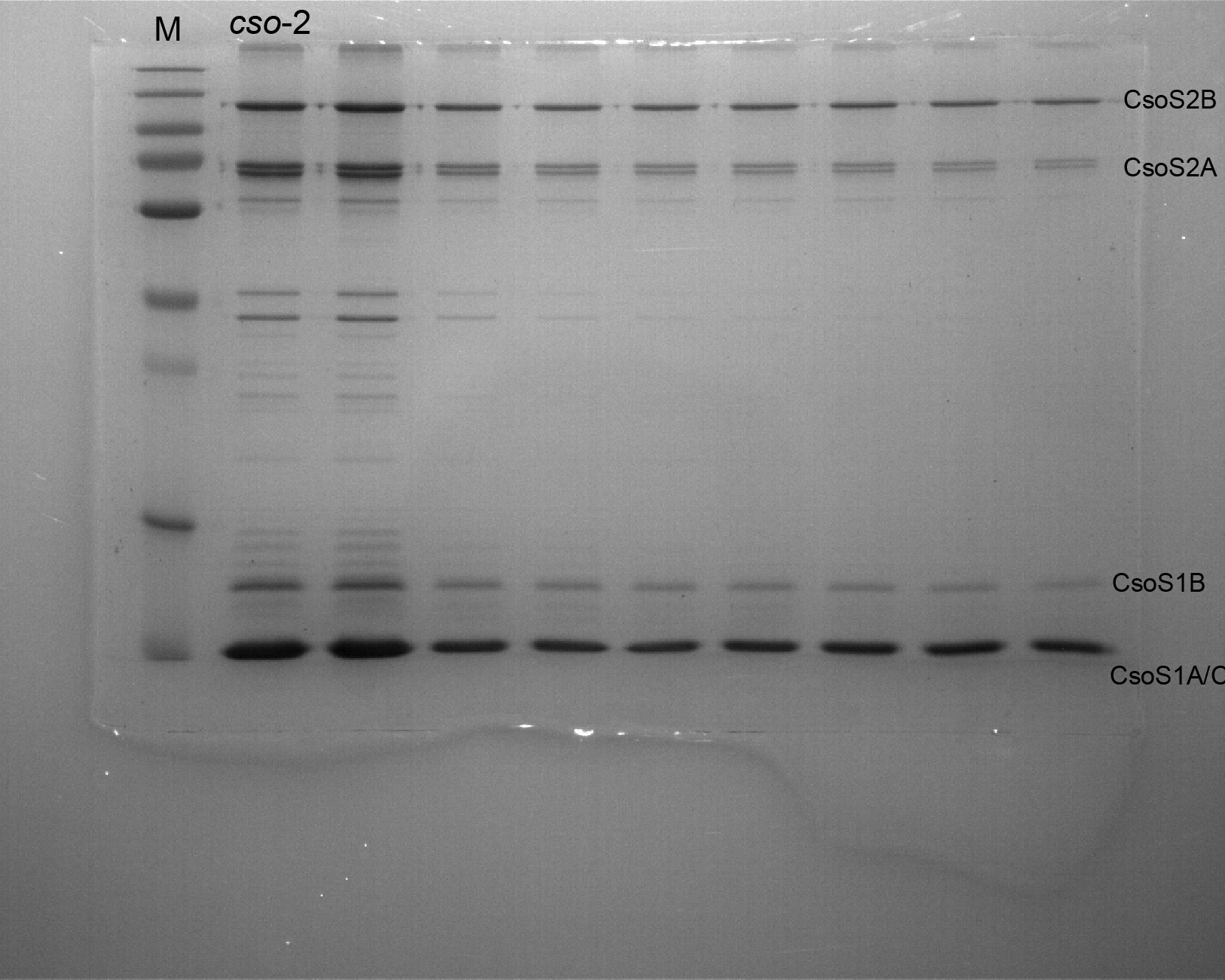

Supplement: Supplementary file 3 — Source data [file 41467_2020_19280_MOESM3_ESM.zip › Source Data_R2/Figure 1/Fig. 1d cso-2 add text.tif]

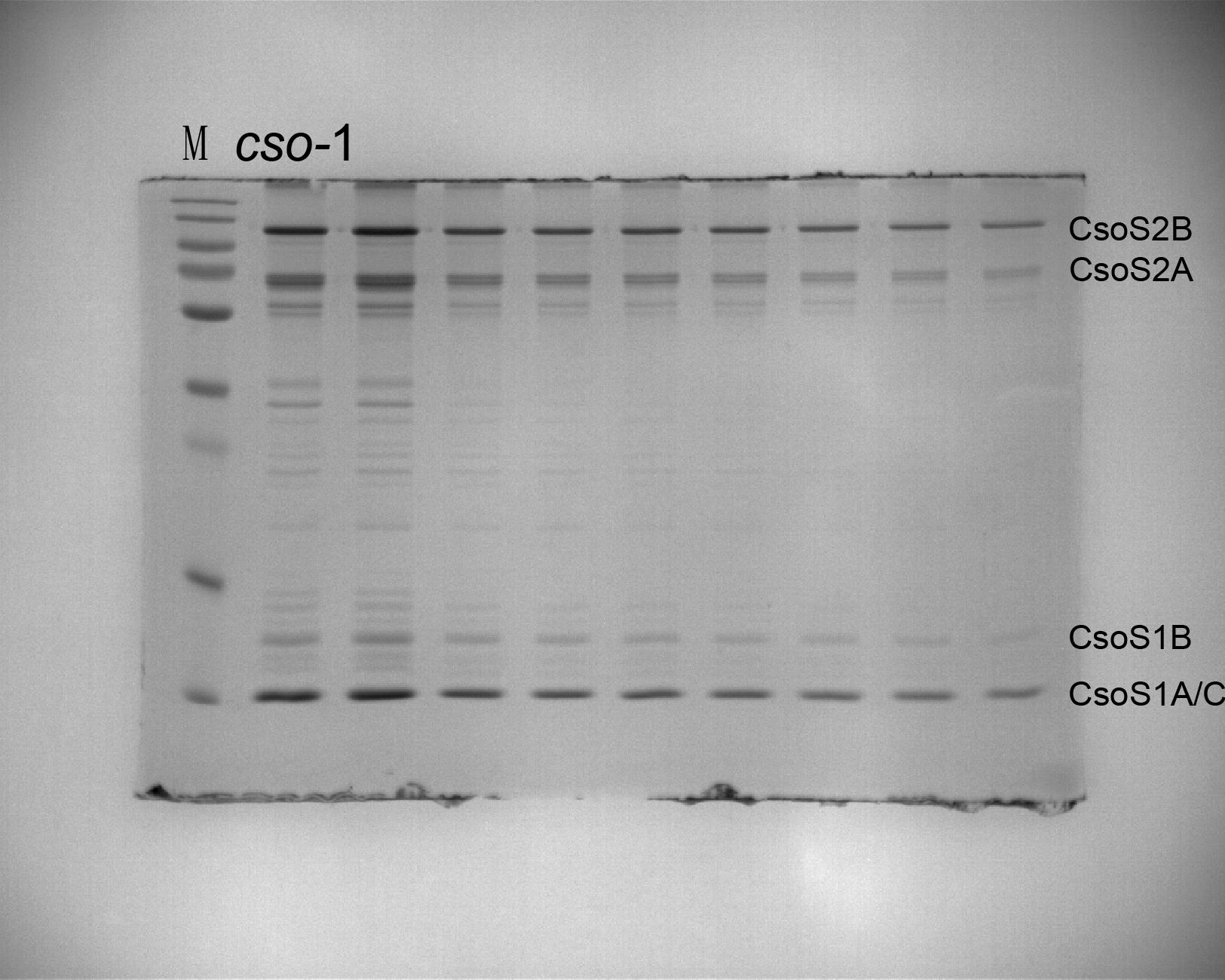

Supplement: Supplementary file 3 — Source data [file 41467_2020_19280_MOESM3_ESM.zip › Source Data_R2/Figure 1/Fig. 1d cso-1 add text.tif]

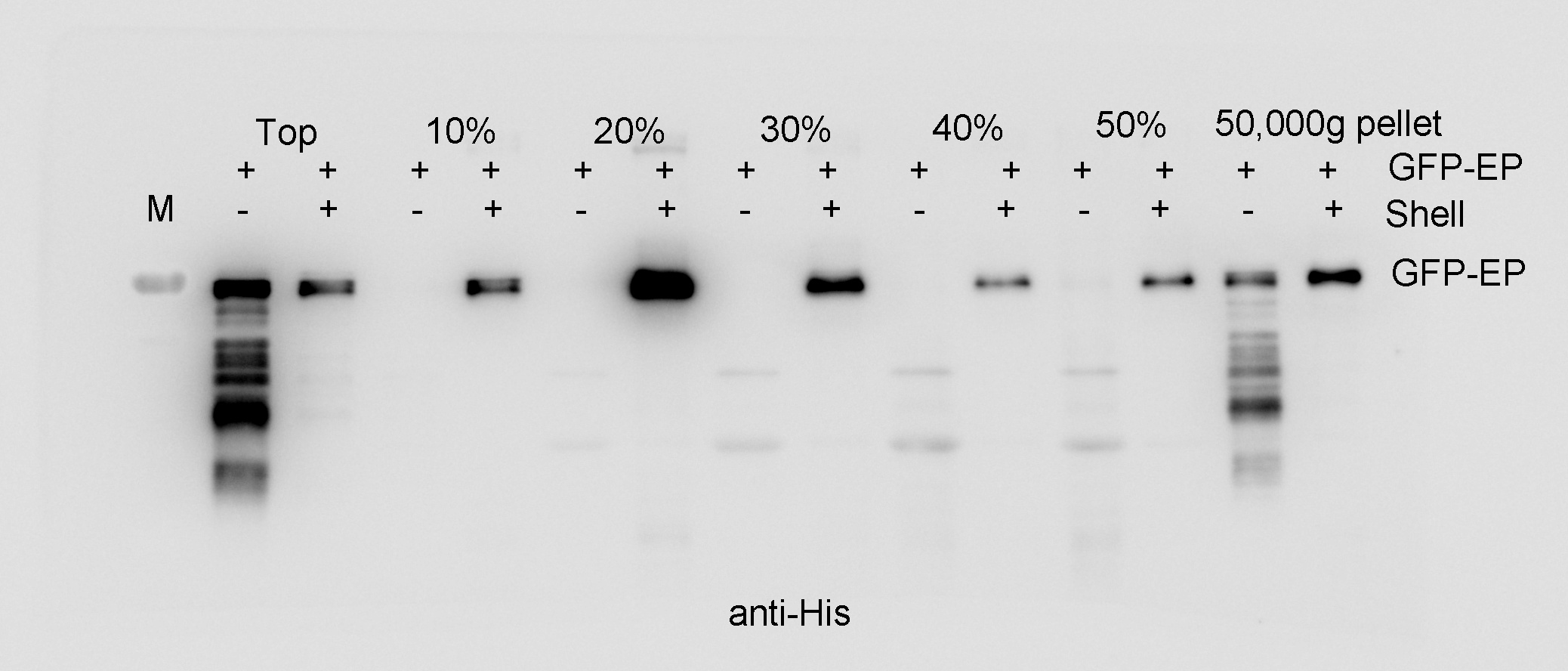

Supplement: Supplementary file 3 — Source data [file 41467_2020_19280_MOESM3_ESM.zip › Source Data_R2/supplementary Fig. 3/anti-His.tif]

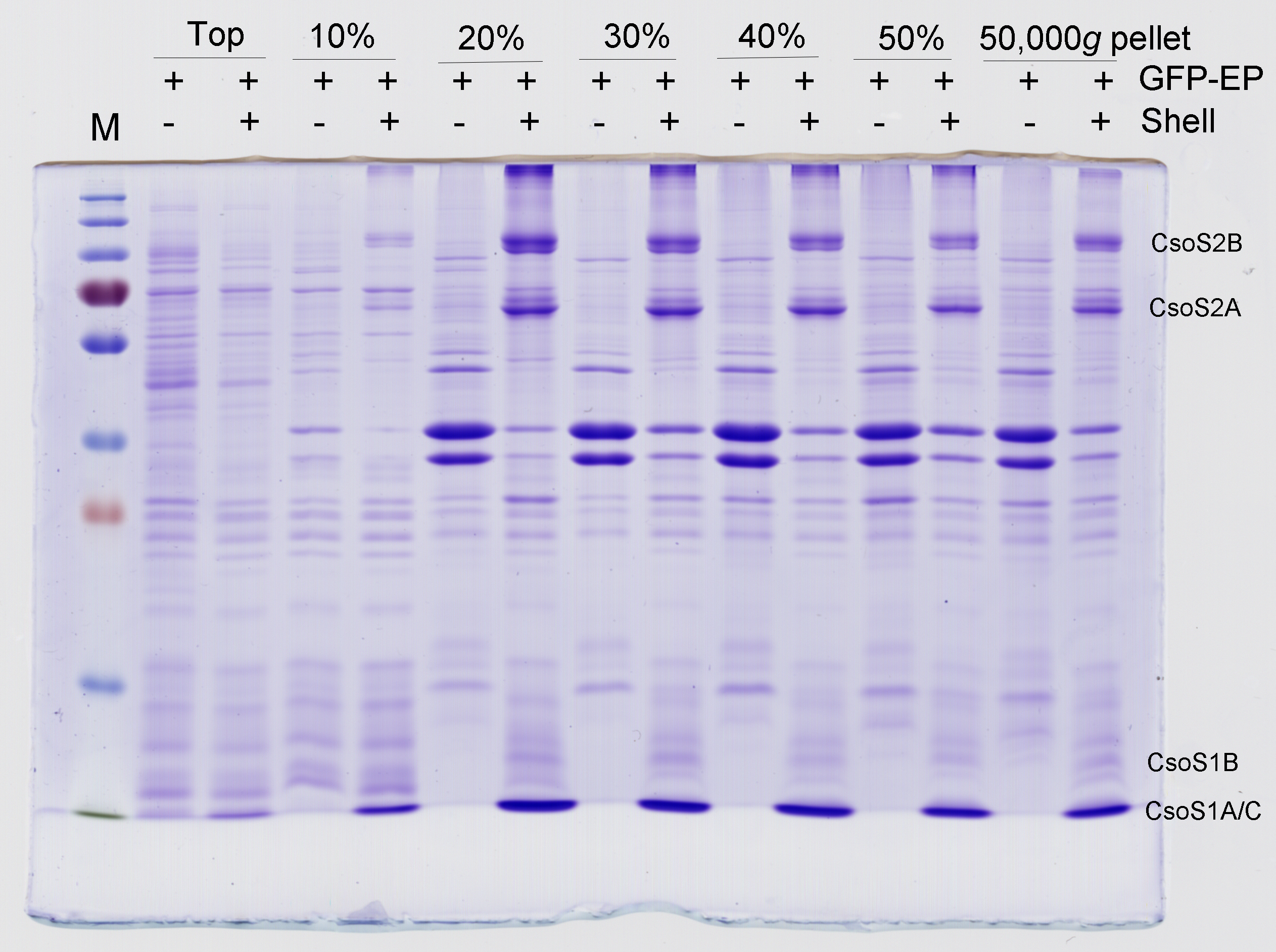

Supplement: Supplementary file 3 — Source data [file 41467_2020_19280_MOESM3_ESM.zip › Source Data_R2/supplementary Fig. 3/coomassie staining.tif]

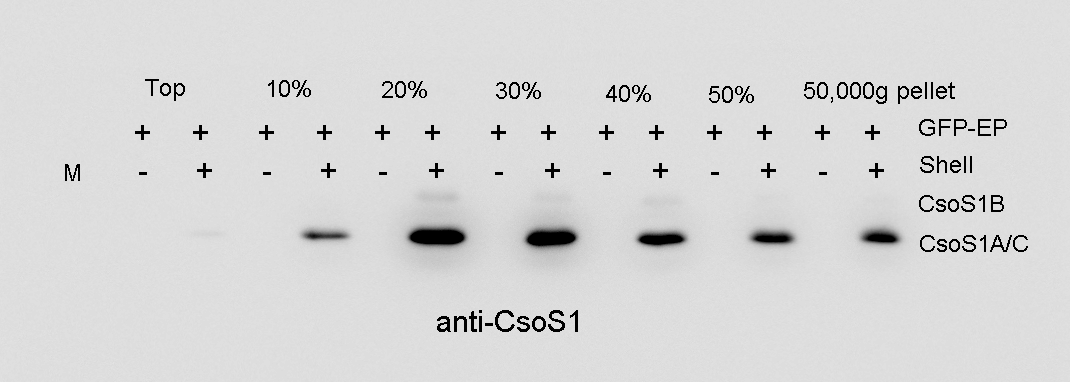

Supplement: Supplementary file 3 — Source data [file 41467_2020_19280_MOESM3_ESM.zip › Source Data_R2/supplementary Fig. 3/anti-CsoS1.tif]

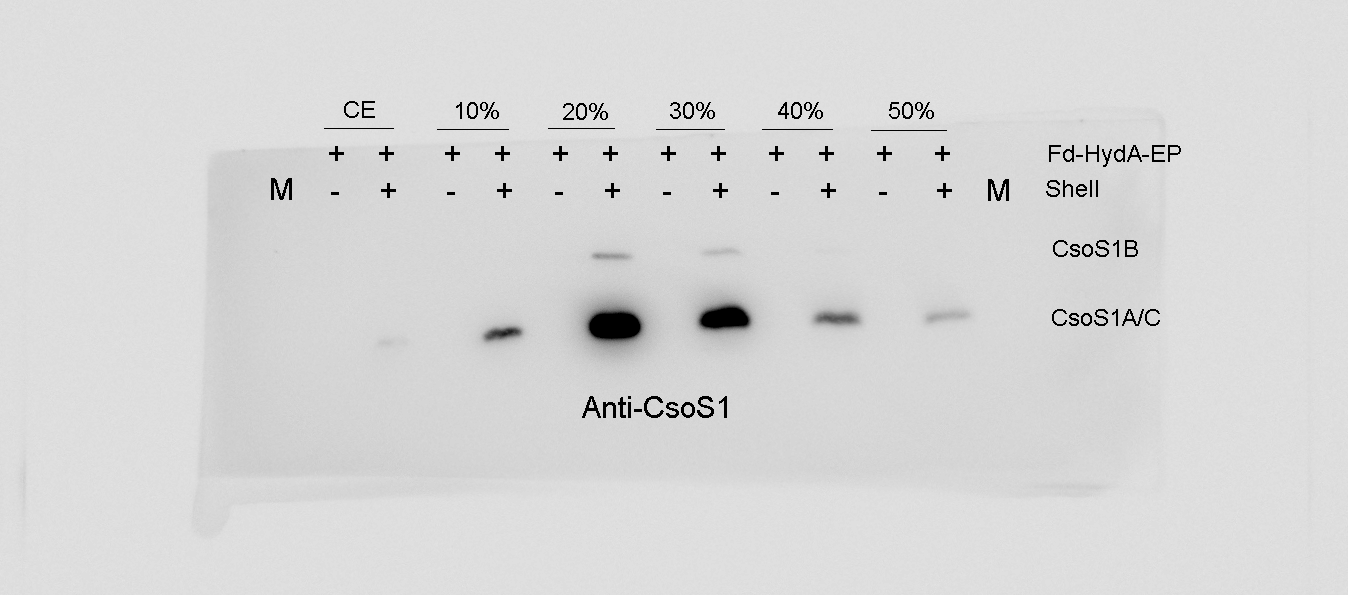

Supplement: Supplementary file 3 — Source data [file 41467_2020_19280_MOESM3_ESM.zip › Source Data_R2/Figure 3/Fig. 3c anti-CsoS1.tif]

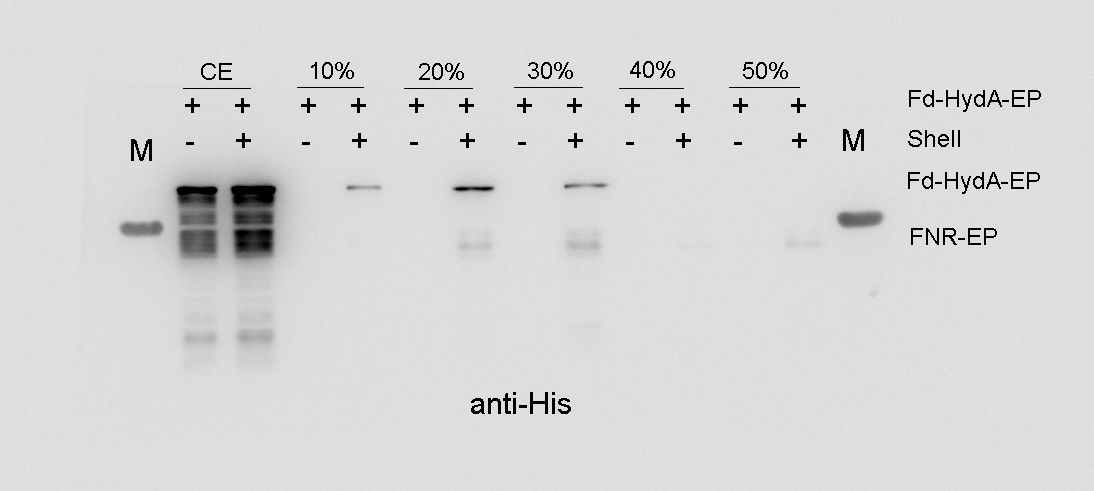

Supplement: Supplementary file 3 — Source data [file 41467_2020_19280_MOESM3_ESM.zip › Source Data_R2/Figure 3/Fig. 3c anti-His.tif]

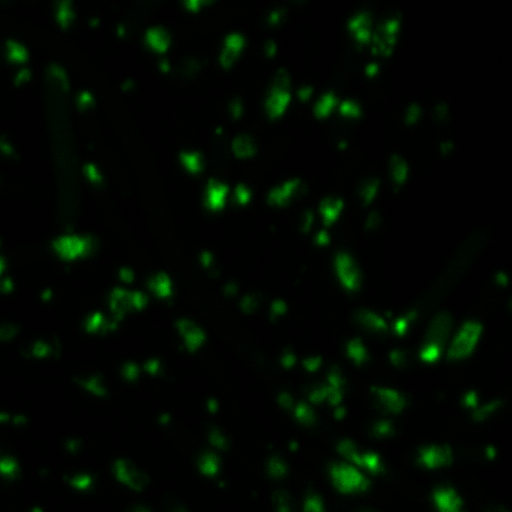

Supplement: Supplementary file 3 — Source data [file 41467_2020_19280_MOESM3_ESM.zip › Source Data_R2/Figure 2/Fig. 2d below.tif]

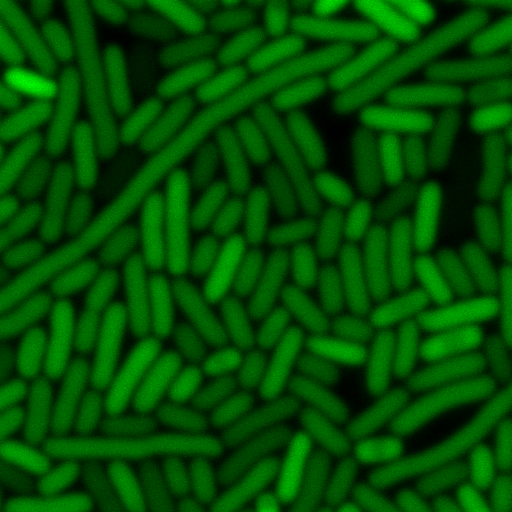

Supplement: Supplementary file 3 — Source data [file 41467_2020_19280_MOESM3_ESM.zip › Source Data_R2/Figure 2/Fig. 2e egfp-CsoS2-C.tif]

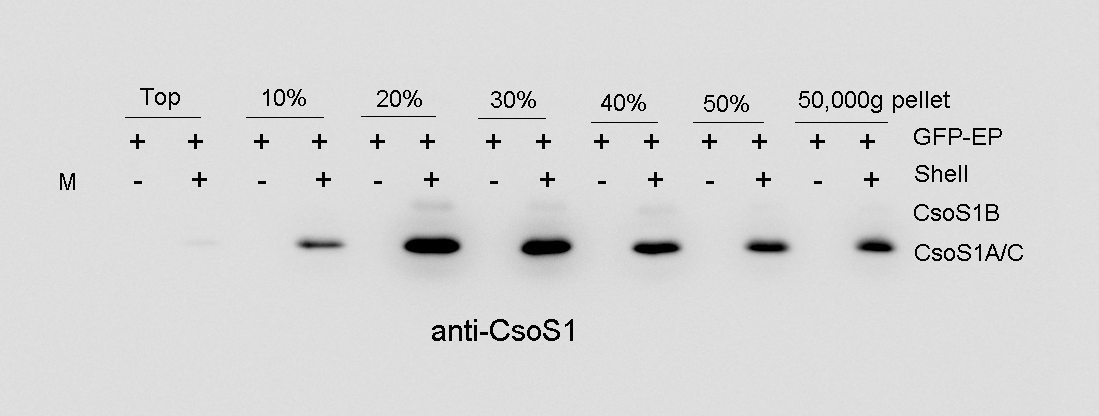

Supplement: Supplementary file 3 — Source data [file 41467_2020_19280_MOESM3_ESM.zip › Source Data_R2/Figure 2/Fig. 2f anti-CsoS1.tif]

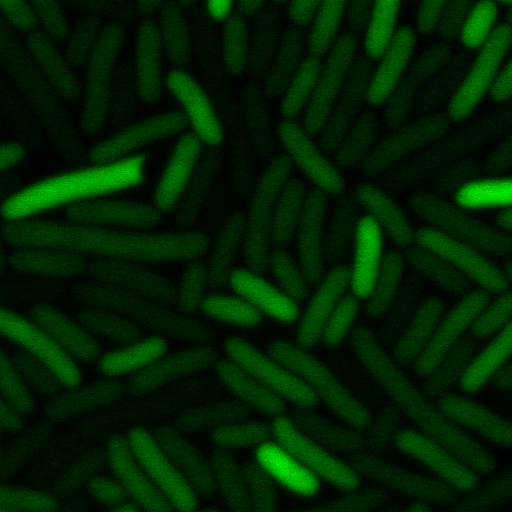

Supplement: Supplementary file 3 — Source data [file 41467_2020_19280_MOESM3_ESM.zip › Source Data_R2/Figure 2/Fig. 2e eGFP and cso-2.tif]

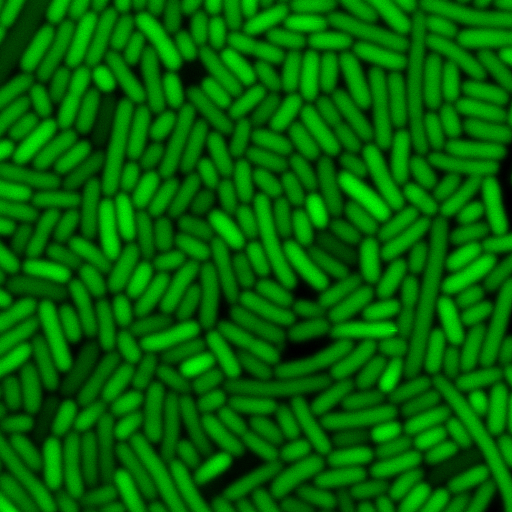

Supplement: Supplementary file 3 — Source data [file 41467_2020_19280_MOESM3_ESM.zip › Source Data_R2/Figure 2/Fig. 2e eGFP.tif]

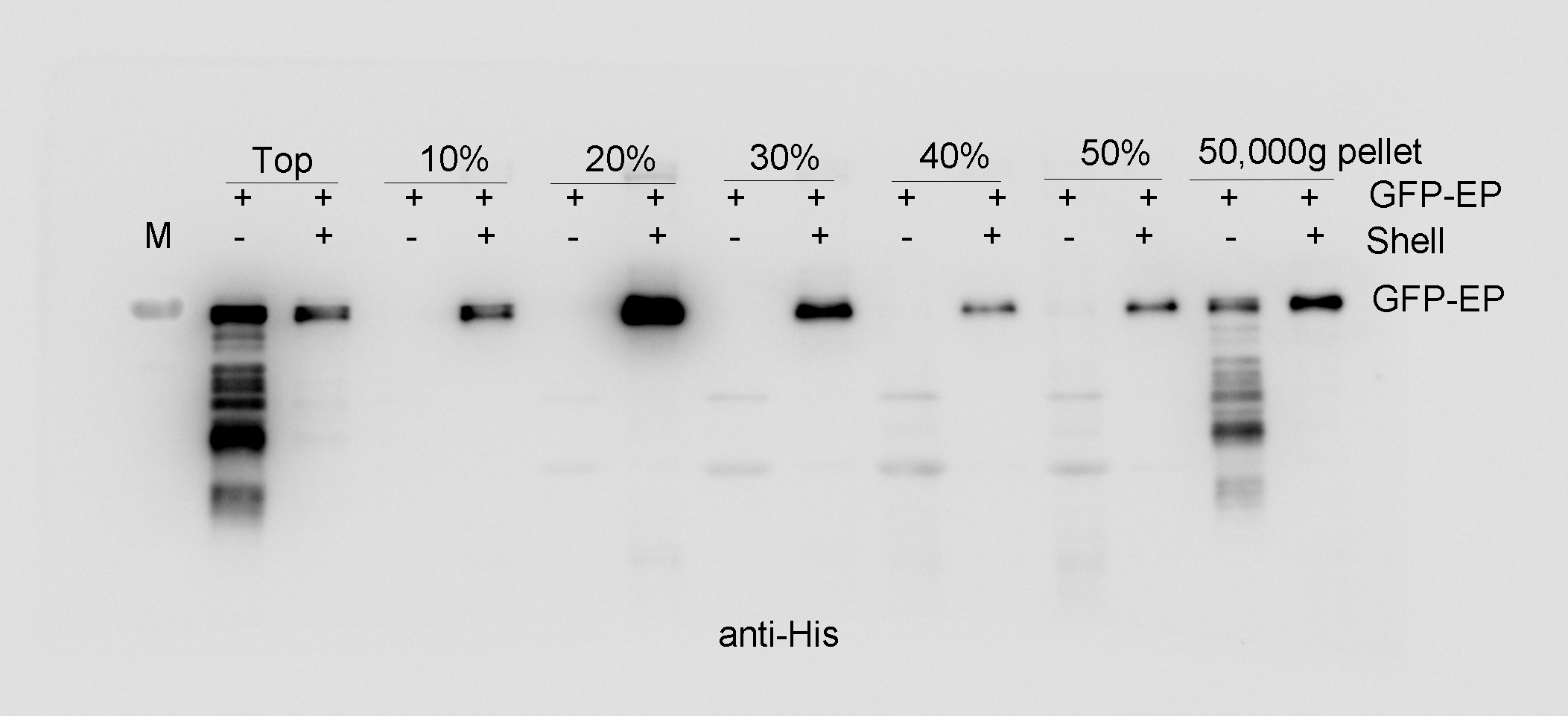

Supplement: Supplementary file 3 — Source data [file 41467_2020_19280_MOESM3_ESM.zip › Source Data_R2/Figure 2/Fig. 2f anti-His.tif]

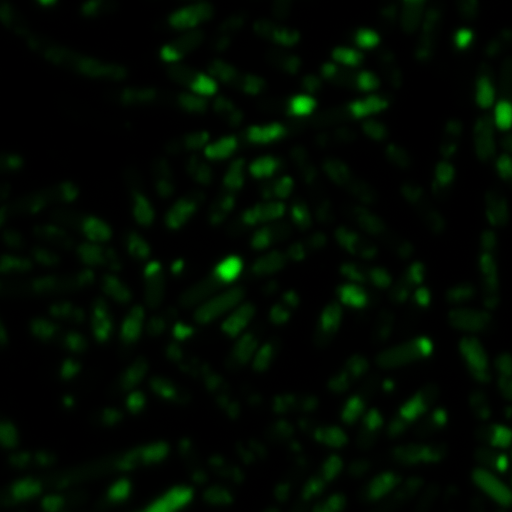

Supplement: Supplementary file 3 — Source data [file 41467_2020_19280_MOESM3_ESM.zip › Source Data_R2/Figure 2/Fig. 2e eGFP-CsoS2-C and cso-2.tif]

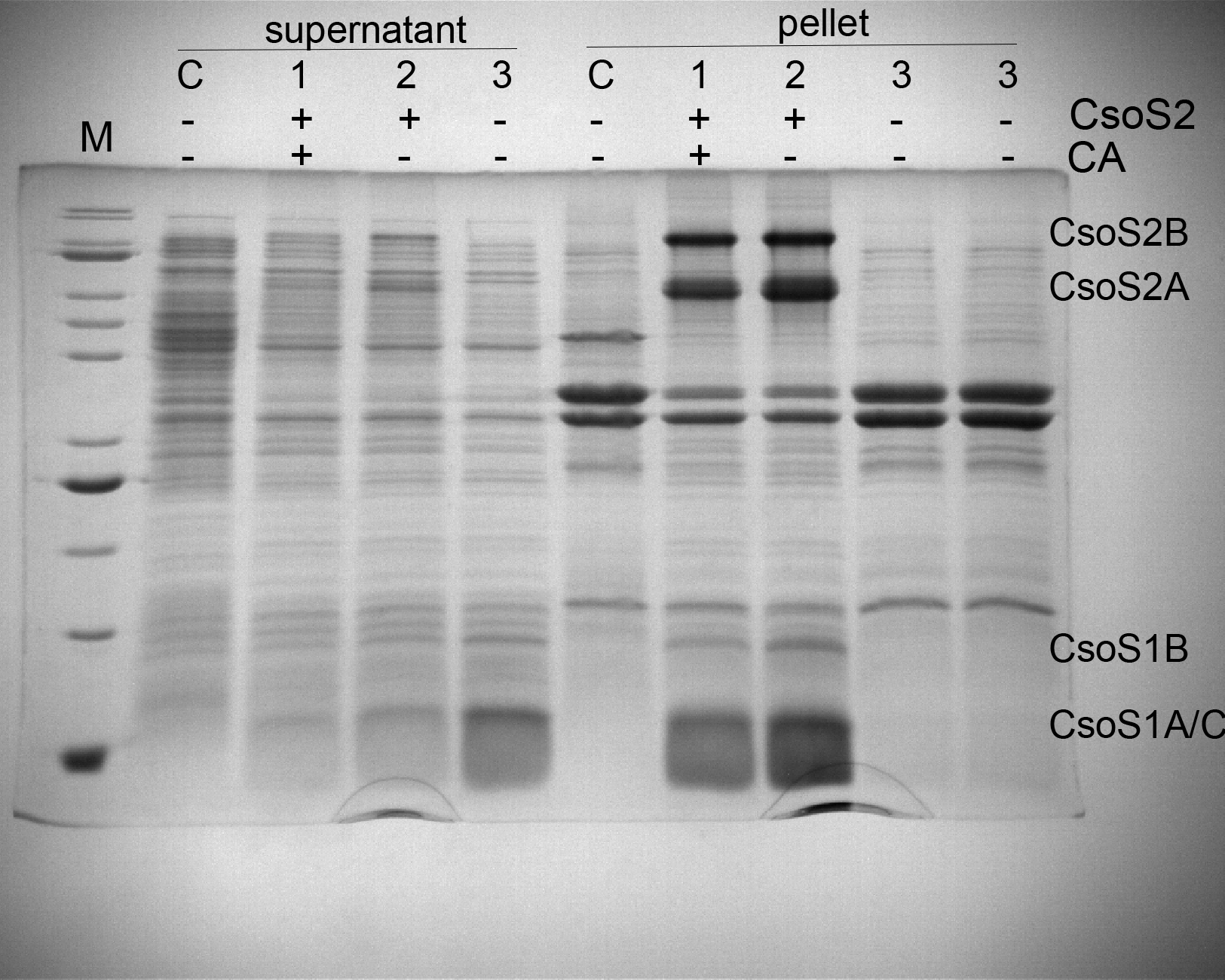

Supplement: Supplementary file 3 — Source data [file 41467_2020_19280_MOESM3_ESM.zip › Source Data_R2/Figure 2/Fig. 2c.tif]

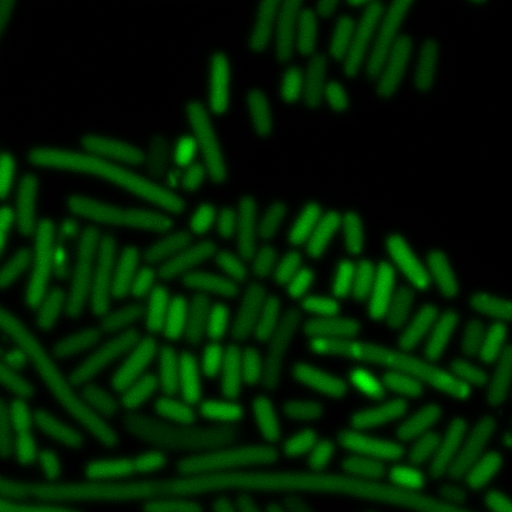

Supplement: Supplementary file 3 — Source data [file 41467_2020_19280_MOESM3_ESM.zip › Source Data_R2/Figure 2/Fig. 2d middle.tif]
